# Carnitine Traffic in Cells. Link With Cancer

**DOI:** 10.3389/fcell.2020.583850

**Published:** 2020-09-18

**Authors:** Lara Console, Mariafrancesca Scalise, Tiziano Mazza, Lorena Pochini, Michele Galluccio, Nicola Giangregorio, Annamaria Tonazzi, Cesare Indiveri

**Affiliations:** ^1^Unit of Biochemistry and Molecular Biotechnology, Department DiBEST (Biologia, Ecologia, Scienze della Terra), University of Calabria, Arcavacata di Rende, Italy; ^2^Institute of Biomembranes, Bioenergetics and Molecular Biotechnologies (IBIOM), National Research Council, Bari, Italy

**Keywords:** transporters, SLC, mitochondria, β-oxidation, carnitine, cancer, drugs

## Abstract

Metabolic flexibility is a peculiar hallmark of cancer cells. A growing number of observations reveal that tumors can utilize a wide range of substrates to sustain cell survival and proliferation. The diversity of carbon sources is indicative of metabolic heterogeneity not only across different types of cancer but also within those sharing a common origin. Apart from the well-assessed alteration in glucose and amino acid metabolisms, there are pieces of evidence that cancer cells display alterations of lipid metabolism as well; indeed, some tumors use fatty acid oxidation (FAO) as the main source of energy and express high levels of FAO enzymes. In this metabolic pathway, the cofactor carnitine is crucial since it serves as a “shuttle-molecule” to allow fatty acid acyl moieties entering the mitochondrial matrix where these molecules are oxidized via the β-oxidation pathway. This role, together with others played by carnitine in cell metabolism, underlies the fine regulation of carnitine traffic among different tissues and, within a cell, among different subcellular compartments. Specific membrane transporters mediate carnitine and carnitine derivatives flux across the cell membranes. Among the SLCs, the plasma membrane transporters OCTN2 (Organic cation transport novel 2 or SLC22A5), CT2 (Carnitine transporter 2 or SLC22A16), MCT9 (Monocarboxylate transporter 9 or SLC16A9) and ATB^0, +^ [Sodium- and chloride-dependent neutral and basic amino acid transporter B(0+) or SLC6A14] together with the mitochondrial membrane transporter CAC (Mitochondrial carnitine/acylcarnitine carrier or SLC25A20) are the most acknowledged to mediate the flux of carnitine. The concerted action of these proteins creates a carnitine network that becomes relevant in the context of cancer metabolic rewiring. Therefore, molecular mechanisms underlying modulation of function and expression of carnitine transporters are dealt with furnishing some perspective for cancer treatment.

## Introduction

**FIGURE 1:**
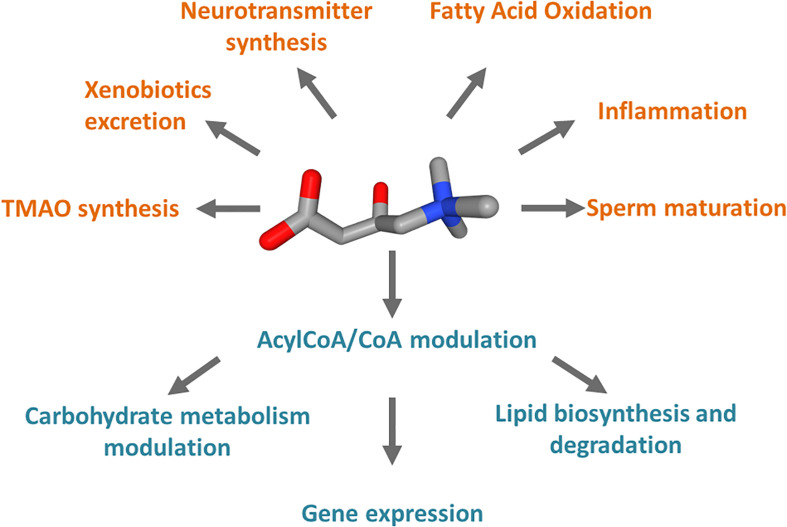
Schematic representation of the cell processes linked to carnitine. Carnitine is represented as 3D conformer with oxygen atoms in red and ammonium atom in blue. In orange, pathways in which carnitine is involved and compounds synthesized from carnitine metabolism. In teal, processes regulated by the Acyl-CoA/CoA ratio derived from carnitine function in mitochondria.

Carnitine is a crucial cofactor given its pleiotropic role in human metabolism (Figure 1). The endogenous biosynthesis which mainly takes place in the liver, kidney, and to some extent in the brain, meets only 25% of the carnitine required by the human body, while the remaining 75% is obtained from the diet under regular diet regimen, i.e., consuming either meat, fish, dairy product, and vegetables (Longo et al., 2006; Almannai et al., 2019). Therefore, carnitine homeostasis is maintained by the balance between intestinal absorption, endogenous synthesis, and renal reabsorption (Pochini et al., 2013; Almannai et al., 2019). In particular, the kidney needs to be very efficient in regulating urinary carnitine excretion; in case of a strict vegetarian diet, the renal reabsorption, together with an increase of biosynthesis, compensate, at least partially, for inadequate carnitine intake (Lombard et al., 1989; Vaz and Wanders, 2002; Rebouche, 2004). Carnitine homeostasis does not consist of the simple maintenance of constant carnitine concentration. Cells from various tissues require different amounts of carnitine to ensure their survival; as an example, the highest carnitine level in the human body is reached in testis where carnitine is necessary for sperm maturation (Bresolin et al., 1982; Rebouche and Seim, 1998; Pochini et al., 2019). A proof of the dynamic balancing of carnitine homeostasis derives from the observation that, under regular diet, patients carrying defects of the carnitine biosynthesis enzymes, do not apparently display carnitine deficiency, because the reduced synthesis is compensated by carnitine dietary intake and increased renal reabsorption (El-Hattab and Scaglia, 2015). On the contrary, defects of the transport protein that mainly mediated the absorption and renal reabsorption of carnitine, lead to a syndrome called primary carnitine deficiency (PCD – OMIM 212140; Stanley et al., 1992; Magoulas and El-Hattab, 2012; Rose et al., 2012). The maintenance of carnitine homeostasis is crucial in cell metabolism due to the major role of carnitine as a shuttle of acyl groups for fatty acid oxidation (FAO). Indeed, carnitine can be converted into acylcarnitine by acyltransferase isoenzymes present in various subcellular compartments. As an example, in the heart acylcarnitine may act as a reservoir of activated acyl groups that can be transferred to CoA to provide an immediate source of energy through FAO. It has to be stressed that several features make carnitine an optimal vehicle for moving acyl groups: at first, the carnitine derivatives are more stable and less reactive with respect to the acyl-CoA (Ramsay and Arduini, 1993); moreover, acylcarnitine can be moved across plasma as well as intracellular membranes by specific transporters, shuttling the acyl groups among the different subcellular compartments to meet the metabolic needs (Indiveri et al., 2010). For instance, the mitochondrial “shuttle-system” allows the transport of fatty acids, as acylcarnitines, from the cytosol into the mitochondrial matrix where these nutrients are oxidized for ATP production. Similar shuttles have been proposed in peroxisomes or the endoplasmic reticulum (ER), but definitive demonstrations of their existence are still lacking (Tonazzi et al., 2006; Pochini et al., 2013; Demarquoy and Le Borgne, 2015; Juraszek and Nalecz, 2019). Carnitine is then involved in the regulation of the acyl-CoA/CoA balance that has important consequences in the modulation of carbohydrate metabolism, lipid biosynthesis and degradation, and gene expression (Figure 1; Pietrocola et al., 2015). For instance, a high acetyl-CoA/CoA ratio in the mitochondrial matrix inhibits pyruvate dehydrogenase (PDH) that catalyzes the oxidative decarboxylation of pyruvate to acetyl-CoA (Pietrocola et al., 2015). Noteworthy, PDH represents a connection between glycolysis and the tricarboxylic acid (TCA) cycle; then, a decrease of acetyl-CoA/CoA ratio can relieve the inhibition of PDH thus affecting glucose oxidation. On the contrary, Acetyl-CoA is an activator of pyruvate carboxylase, thus promoting the TCA flux or the gluconeogenesis (Pietrocola et al., 2015). These allosteric effects can be modulated through the action of carnitine acetyltransferase (CAT), a mitochondrial isoenzyme that transfers the acetyl groups from acetyl-CoA to carnitine, forming acetylcarnitine. In good agreement with the above-described processes, carnitine seems to be involved also in insulin sensitivity (Figure 1): it was observed that the muscle accumulation of fatty acyl-CoA derivatives or acyl metabolites inhibits both insulin signaling and glucose oxidation (Pietrocola et al., 2015; Console et al., 2018). Another metabolically relevant compound that links carnitine and CoA derivatives is the malonyl-CoA that is required for fatty acid biosynthesis but acts as a potent inhibitor of the carnitine acyltransferases using cytoplasmic substrates, such as carnitine palmitoyltransferase 1 (CPT1), which is the first component of the mitochondrial carnitine shuttle (Ramsay and Arduini, 1993). Thus, when the malonyl-CoA concentration rises, the fatty acid synthesis increases but the flow of acylcarnitine into the mitochondrial matrix and, in turn, into the β-oxidation pathway decreases. Furthermore, the acetyl-CoA derived from carnitine has been also reported to regulate gene expression by influencing histone acetylation (Madiraju et al., 2009). A novel role for carnitine was recently discovered as potentially relevant to human health; it was reported that carnitine is a dietary precursor from which gut microbiota releases trimethylamine (TMA) which is then subjected to human cell metabolism and converted to trimethylamine N-oxide (TMAO) by hepatic flavin monooxygenase (FMO; Phillips and Shephard, 2020). In terms of relevance to human health, carnitine shows anti-inflammatory, and anti-oxidant properties (Bene et al., 2018; Wang et al., 2020; Figure 1). In the brain, acetylcarnitine supports the synthesis of neurotransmitters (Pochini et al., 2019; Figure 1). Finally, carnitine is also used to reduce the toxicity of compounds deriving from partially metabolized acyl groups and breakdown of xenobiotics by facilitating their excretion in carnitine ester form (Duran et al., 1990; Bene et al., 2018; Figure 1). The wide collection of functions above described, could not be met without a proper interconnection across the tissues and the cell sub-compartments provided by dedicated membrane transporters that regulate the carnitine traffic in the human body. In this respect, it is not surprising that under pathological conditions such as cancer, this traffic is altered. An overview of the relationships among alterations of carnitine traffic and the metabolic switch typical of cancer cells will be the object of the present review.

## Carnitine Traffic

**FIGURE 2:**
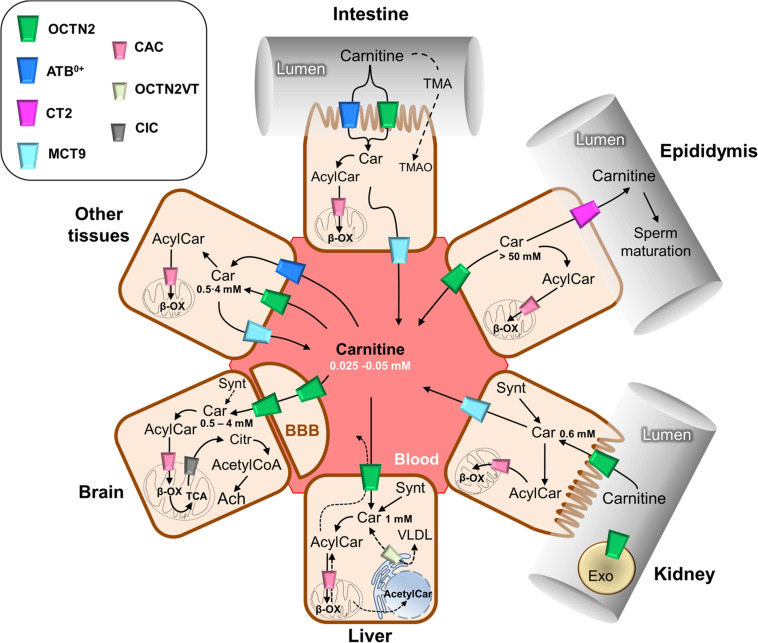
Schematic representation of carnitine traffic. Carnitine (Car) traffic is described by continuous arrows; dotted arrows refer to the transport of some other substrates such as carnitine derivatives and trimethylamine (TMA) that are indirectly involved in carnitine traffic. The organic cation transporter novel 2 (OCTN2) mediates carnitine transport by a sodium dependent mechanism, which is not reported for the sake of clarity. ATB^0,+^ mediates a sodium and chloride dependent transport, which is not reported for the sake of clarity. Carnitine/acylcarnitine carrier (CAC), the mitochondrial carnitine transporter, allows for the completion of β-oxidation (β-ox) and mediates acylcarnitine/carnitine antiport in mitochondria which is reported in details in Figure 3. The longest isoform of OCTN2, named OCTN2VT, is depicted in the endoplasmic reticulum (ER). The other transporters are depicted in the cell types in which they are expressed. The role of carnitine in the synthesis of the neurotransmitter acetylcholine (Ach) is reported in the sketch representing the brain tissue; the blood-brain barrier is indicated as BBB. Carnitine concentrations in the different tissues are reported. Synt, carnitine synthesis; CIC, citrate transporter; Citr, citrate; Exo, exosomes; VLDL, very low density lipoprotein; BBB, blood-brain barrier.

**FIGURE 3:**
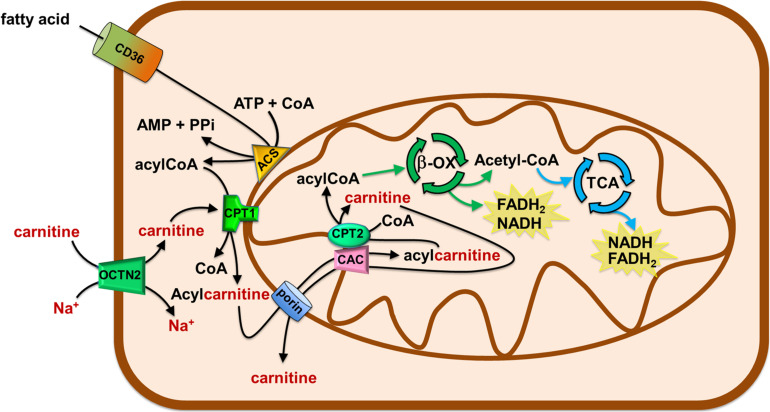
Role of Carnitine in the mitochondrial β-oxidation pathway. The organic cation transporter novel 2 (OCTN2) mediates the uptake of carnitine in cells. Fatty acids (FA) crosses the plasma membrane via CD36. In the cytosol, FA are converted in acyl-CoA by acetyl-CoA synthetase (ACS) and then are translocated in the mitochondrial matrix by the carnitine shuttle. The shuttle is constituted by carnitine palmitoyltransferase 1 (CPT1), carnitine/acylcarnitine carrier (CAC), and carnitine palmitoyltransferase 2 (CPT2). Once in the matrix, acyl-CoA undergoes β-oxidation (β-ox) with the production of acetyl-CoA that enters the tricarboxylic acid cycle (TCA). NADH and FADH_2_ are generated by β-oxidation and TCA.

The carnitine traffic in the body is regulated by a dedicated network of membrane transport proteins showing different tissue and subcellular localization (Figure 2). The intestinal absorption of carnitine, as well as its renal reabsorption, is primarily mediated by the plasma membrane organic cation transporter novel 2 (OCTN2 – SLC22A5) that, among the carnitine-handling transporters, shows the highest affinity toward carnitine. OCTN2 plays a major role also in the distribution of carnitine to the various tissues (Figure 2). Besides intestine and kidney, OCTN2 is expressed in several tissues such as placenta, mammary gland, liver, heart, testis, skeletal muscle, and brain, where it is also expressed at the level of the blood-brain barrier (BBB; Koepsell, 2013; Pochini et al., 2019; Koepsell, 2020). In good agreement with the localization of OCTN2 in the brain, the administration of carnitine-derivatives may sustain neuroprotection (Juraszek and Nalecz, 2019; Pochini et al., 2019). The carnitine transport mediated by OCTN2 is Na^+^-dependent. This feature allows for carnitine accumulation in cells giving rise to a concentration gradient between intracellular space and blood. The intracellular concentration of carnitine ranges from 1 to 5 mM, while the concentration in the plasma ranges from 25 to 50 μM (Bene et al., 2018). The major role of OCTN2 in carnitine absorption and tissue distribution is demonstrated by the existence of a human disease caused by inborn defects of the OCTN2 gene, namely PCD (Magoulas and El-Hattab, 2012). The disease is characterized by general metabolic derangement, cardiomyopathy, hyperammonemia, hypoglycemia, muscle weakness, and myopathy, in line with the crucial role of carnitine in FAO (Nezu et al., 1999). Interestingly, the clinical manifestations of the disorder can be improved by supplementation of high doses of carnitine. The partial rescue of the above-described symptomatology can be ascribed to the activity of other transporters such as ATB^0,+^(SLC6A14), MCT9, and probably, OCTN1 (SLC22A4) that accept carnitine with a much lower affinity if compared to OCTN2. In particular, the relevance of the ATB^0,+^-mediated carnitine transport, in patients with PCD, may be explained considering that this protein is highly expressed in the intestine (Figure 2). ATB^0,+^ is also expressed in the lung (Sloan et al., 2003), eye (Ganapathy and Ganapathy, 2005), and mammary gland (Nakanishi et al., 2001) allowing for carnitine distribution in these tissues. Concerning the plasma membrane transporter MCT9, its transport features are still poorly characterized. MCT9 is ubiquitous with the highest expression level in the kidney and adrenal gland; then, its involvement in managing carnitine traffic is plausible (Figure 2; Halestrap and Wilson, 2012; Halestrap, 2013). Indeed, MCT9 in the basolateral membrane of enterocytes might ensure the passage of carnitine into the blood. However, no conclusive data are available on its involvement in carnitine traffic under physiological conditions. MCT9 is also expressed in human umbilical vein endothelial cells where it may have a role in the pro-inflammatory response linked to carnitine (Figure 1). Indeed, the MCT9 expression is increased by the pro-inflammatory tumor necrosis factor-α (TNF-α); this, as a consequence, induces a carnitine accumulation in endothelial cells contributing to energy production via FAO for sustaining the inflammatory response (Knyazev et al., 2018). Even more controversial is the role played by OCTN1 that is ubiquitously expressed even if its actual physiological role in carnitine homeostasis is uncertain. There is evidence on its ability to mediate carnitine and acetylcarnitine transport albeit with a very low affinity (Pochini et al., 2011, 2016) since carnitine is not its main substrate. One of the physiological substrates of this transporter is acetylcholine, underlying a key role in the non-neuronal cholinergic system in both physiological and pathological conditions, as testified by the occurrence of a natural mutation of OCTN1 in inflammatory conditions such as Crohn’s Disease (Pochini et al., 2019). An intriguing history is related to another member of the OCTN family, namely OCTN3 (SLC22A21). It is a carnitine specific transporter that disappeared during evolution: it is present in mouse, but not in chimpanzee or humans (Pochini et al., 2013). As stated in the introduction, carnitine homeostasis is also gender-specific, being crucial for sperm maturation (Figures 1, 2). In testis and the epididymis, carnitine concentration reaches the highest level of the body, up to 60 mM in the epidydimal lumen. The terrific carnitine concentration gradient between epidydimal lumen and plasma is allowed by the presence of OCTN2 at the blood side and by the presence of another carnitine specific transporter, namely CT2 (SLC22A16) on the lumen side of epithelia (Gong et al., 2002). The functional characterization of CT2 is still at its infancy, but its peculiar tissue distribution suggests that this transporter is involved in maintaining the epidydimal gradient of carnitine that plays as osmolyte and FAO cofactor. Surprisingly, CT2 becomes almost ubiquitous in human cancers with a dramatic broadening of its function (Koepsell, 2013). Another tissue with high content of carnitine is placenta. In the mouse placenta carnitine concentration is up to 10-fold compared with heart. Also in this tissue, the transport of carnitine is mediated by OCTN2 (Shekhawat et al., 2004). The placenta has to provide the fetus with the nutrients and to serve to eliminate waste products of fetal metabolism. To perform these unique roles placenta requires energy. At first it was established that glucose was the major source of energy for the placenta-fetus unit, but more recent studies demonstrated that FAO play an important role for placental and fetus growth (Shekhawat et al., 2004). Moreover, carnitine seems to be critical in fetal maturation, and fetal gene regulation. Studies also have showed a correlation between the intrauterine growth retardation due to hypoglycemia and to hypoxia-ischemia in the newborn with decreased plasma free carnitine concentration and impaired FAO (Xi et al., 2008). After entering the cell, carnitine traffic among the cytosol and the intracellular compartments is needed. Therefore, transporters located in organelle membranes must operate to fulfill the intracellular traffic of carnitine and its derivatives. The most well-studied transporter of the endocellular membranes is the mitochondrial carnitine/acylcarnitine carrier (CAC; SLC25A20) that is the central component of the mitochondrial carnitine shuttle (Figure 3). This system includes the CPT1 which grapples on the mitochondrial outer membrane and converts acyl-CoAs into acylcarnitines. These cross the inner mitochondrial membrane through the CAC. Once in the mitochondrial matrix, carnitine palmitoyltransferase 2 (CPT2) converts acylcarnitines back into acyl-CoAs for oxidation and energy production (Figure 3; Indiveri et al., 2011). The essential link between the mitochondrial carnitine transport and FAO is demonstrated by the life-threatening inherited carnitine/acylcarnitine translocase deficiency (OMIM 613698) that, in contrast to the PCD caused by OCTN2 defects, cannot be compensated by the administration of carnitine (Indiveri et al., 2011). Less information is available on carnitine transporters of other subcellular compartments such as the ER. The longest splicing variant of the SLC22A5 gene codes for an OCTN2 variant called OCTN2VT containing an insertion of 24 amino acids in the first extracellular loop, that seems to be localized in the ER (Figure 2; Maekawa et al., 2007; Pochini et al., 2013). A still unsolved issue, connected with the role of carnitine in modulating gene expression (Figure 1) is the link between mitochondria and nucleus: acetylcarnitine produced in mitochondria would reach the nucleus providing acetyl units (Figure 2). This further step in carnitine traffic can be possible if a nuclear isoform of CAT exists for regenerating acetyl-CoA from acetylcarnitine. This aspect is, indeed still mysterious even if a protein with CAT feature has been described in the nuclear extract from HEK293 cells (Madiraju et al., 2009). Furthermore, the role of carnitine in mediating TMAO synthesis has relevance to human health. Indeed, TMAO was suggested as one of the factors promoting atherosclerosis and increased cardiovascular risk (Koeth et al., 2013; Gao et al., 2017; Roncal et al., 2019). Later on, it has been shown that more than 200 variants exist in the coding region of TMAO producing enzyme, with different outcomes on the cardiovascular risk (Phillips and Shephard, 2020). The actual interest around TMAO and, hence, carnitine contribution to its synthesis, is the association with increased risk of colorectal and gastrointestinal cancers; however, given that TMAO levels are also greatly influenced by diet-microbiota interactions that are subjected to great variability, the link between TMAO and some human cancers cannot be easily defined (Oellgaard et al., 2017). Finally, a novel branch in the carnitine traffic has been suggested which is linked to vesicles of endocellular origin, namely exosomes. In this respect, exosomes loaded with the plasma membrane transporter OCTN2 can transfer from one cell to another, the capacity of absorbing carnitine. In particular, exosomes derived from HEK293 cells treated with interferon-γ (INF-γ) carry a higher amount of OCTN2 with respect to exosomes derived from untreated cells. This correlates well with the suggestion that carnitine is involved in the inflammatory response and that the expression of OCTN2 in epithelia can be modulated by inflammatory cytokines such as INF-γ and TNF-α (Console et al., 2018; Juraszek and Nalecz, 2019; Pochini et al., 2019).

## Fatty Acid Metabolism, Carnitine and Cancer

The carnitine supply is *conditio sine qua non* to carry out the β-oxidation of fatty acids by mitochondria. This is one of the most efficient energy-producing pathways in cells, therefore, high energy demanding tissues, such as heart and kidney, mainly rely on fatty acids utilization (Console et al., 2020). If we consider human diseases, a pathological condition characterized by a high energy request is cancer. Cancer cells need the energy to sustain the high rate of proliferation and the energy need further increases with the grade of malignancy (Hanahan and Weinberg, 2011). The association of FAO derangements with cancer has been studied since 1952 (Waterman et al., 1952). However, the current largest body of evidence, in the field of metabolic adaptation of cancer cells, deals with the flexible utilization of glutamine and glucose as the main sources of energy. Glucose and glutamine as energetic substrates are considered a hallmark of cancer cells and the metabolic switch that allow their utilization under virtually anaerobic conditions is known as the Warburg effect (Warburg et al., 1927). The canonical interpretation of the Warburg effect implies that cells bypass the mitochondrial respiratory chain for the synthesis of ATP even in the presence of adequate oxygen supply (Ganapathy et al., 2009). However, it is nowadays evident that the Warburg effect needs to be considered in a more general metabolic context that includes also the utilization of fatty acids in line with the efficiency of these substrates in terms of ATP yield. In this view, the mitochondrial function in cancer is not totally impaired and the TCA, as well as the oxidative phosphorylation pathway, are working (Currie et al., 2013). In agreement, it is more and more evident that some cancers with dual capacity for glycolytic and oxygen-consuming metabolism exist. The described metabolic flexibility is a relevant phenomenon observed in different types of cancers, and, within the same cancer type, at different stages of progression. Concerning the lipid metabolism, there is compelling evidence showing that in some cancer types the fatty acid utilization is increased, while in others this pathway is down-regulated. Noteworthy, in all the mentioned cases, an appropriate intervention in regulating the carnitine level and/or traffic is required, given its role in FAO. The alterations of FAO can affect the availability of membrane structural lipids, the abundance of lipids with signaling functions, the synthesis and degradation of lipids for energy production and utilization (Carracedo et al., 2013; Currie et al., 2013; Rohrig and Schulze, 2016; Koundouros and Poulogiannis, 2020). As an example, prostate cancer and diffuse large B-cell lymphoma, use FAO as the main source of energy and express FAO enzymes at high levels (Liu, 2006; Svensson et al., 2016; Yamamoto et al., 2020). FAO substrates are derived from the external environment via specific transporters able to mediate fatty acid uptake, such as CD36 (Figure 3). This is a membrane transporter allowing for fatty acids storage in adipose tissues and for the uptake of fatty acids in cells to produce ATP. CD36 is a heavy glycosylated protein with a hairpin conformation. Indeed it consists of two transmembrane domains, a large extracellular domain, and two short intracellular domains that are required for CD36 function after substrate binding (Su and Abumrad, 2009). In those cancers relying on lipid metabolism, CD36 and other fatty acid binding proteins (FABP) are over-expressed to catch fatty acids stored in surrounding adipocytes (Koundouros and Poulogiannis, 2020). CD36 is distinct from the transporters regulating the traffic of carnitine and acylcarnitines, since it handles the hydrophobic fatty acid molecules as such, thus not directly participating to the carnitine network. Another source of lipid for FAO is the lipid droplets, which are commonly formed in cancer cells. Besides the direct advantage in terms of ATP synthesized from fatty acids, FAO is also relevant in managing oxidative stress derived from the electron transport chain activity. Indeed, the end-product of FAO, acetyl-CoA enters TCA and can leave the cycle as isocitrate. This is, then, oxidized by cytosolic isocitrate dehydrogenase 1 (IDH1) to α-ketoglutarate with the production of NADPH required for the detoxification from reactive oxygen species (ROS; Jeon et al., 2012; Qu et al., 2016). Then, α-ketoglutarate can enter back into the TCA for completing the cycle. The strong requirement for NADPH is testified by the presence of diverse pathways, which are activated in cancer cells to fulfill their need. NADPH can derive also from the activity of the malic enzyme and the pentose phosphate pathway. The genes encoding for these enzymes are positively regulated by the oncogene AKT which acts upstream the transcription factor Nrf2. Furthermore, AKT may directly activate the nicotinamide adenine nucleotide kinase (NADK) responsible for the phosphorylation of NADH forming NADPH (Jeon et al., 2012; Currie et al., 2013; Koundouros and Poulogiannis, 2020). However, the involvement of lipid metabolism in cancer is not only linked to the oxidative route; indeed, cancer cells are greatly committed in anabolic metabolism required for generating new building blocks that sustain cell growth and proliferation. In this frame, lipids are in the list of molecules required by proliferating cells being components of cell membranes. Therefore, cancer cells not only use FAO to oxidize fatty acids and to derive energy by mitochondria but they also use acetyl-CoA to endogenously synthesize fatty acids in the cytosol using NADPH as key cofactor for anabolic enzymes (Currie et al., 2013). This is a typical example of a futile cycle activated by cancer cells that is forbidden in the “canonical” biochemistry. Such a condition is centered on the removal of inhibition of the carnitine handling enzyme CPT1 via the specific down-regulation of acetyl-CoA carboxylase 2 (ACC2), with a strong reduction of the malonyl-CoA pool responsible for CPT1 inhibition (Koundouros and Poulogiannis, 2020). In this complex network of metabolic alterations promoting FAO in cancer, the traffic of carnitine can be considered the start-up process. Indeed, FAO cannot occur without a proper carnitine traffic interplay (Figure 2; Melone et al., 2018).

## Role of Carnitine Transporters in Cancers

Changes in expression and/or activity of carnitine transporters in plasma or intracellular membranes are expected, especially in those cancers characterized by either an increased or a decreased utilization of fatty acids. The *status artis* of the link of altered carnitine transporter expression with cancer will be depicted in this section. Each transporter will be dealt with and described in terms of its role in the derangements occurring in cancer cells.

### General Features of OCTN2 (SLC22A5)

The membrane transporter OCTN2 is one of the upstream players of the carnitine network (Figure 2). It is one of the 13 members of the SLC22 family that includes organic cation transporters (OCTs), organic zwitterion/cation transporters novel (OCTNs), and organic anion transporters (OATs). The gene encoding the human isoform of OCTN2 has been annotated and cloned in 1998, in parallel to the murine one (Tamai et al., 1998, 2000; Wu et al., 1998, 1999). It maps in the chromosome 5 in the “inflammatory bowel diseases 5 (IBD)” risk region and is formed by 11 exons giving rise to two variants of different lengths, which have different subcellular localization (Figure 2). Over the years, the function of OCTN2 has been characterized, employing intact cell systems as well as proteoliposomes harboring the native rat and human protein (Tamai et al., 1998, 2000; Lahjouji et al., 2002; Ohashi et al., 2002; Pochini et al., 2004; Console et al., 2018). The main substrates of OCTN2 are carnitine and its acyl derivatives and their transport is coupled to Na^+^ co-transport (Figure 3 and Table 1; Pochini et al., 2013). OCTN2 mediates also the transport of organic cations such as TEA in Na^+^-independent manner. Data from studies using cross species chimeric OCTN2 and mutagenesis demonstrated that the active site for carnitine and that for organic cations overlap but are not identical. Indeed, the mutation P478L of OCTN2, abolished only the carnitine transport activity (Seth et al., 1999). This mutation causes the PCD (Seth et al., 1999). An important model to study the consequences of systemic carnitine deficiency is the homozygous OCTN2 null mouse, in which OCTN2 function is abolished by the missense mutation L352R. In contrast with P478L, this second mutation abrogates both carnitine and organic cations transport. Homozygous OCTN2 null mice showed liver fatty infiltration and hypoglycemia. In absence of carnitine administration these mice die within 3–4 weeks after birth with dilated cardiomyopathy, which is also seen in children with PCD (Seth et al., 1999; Shekhawat et al., 2004). Intriguingly, different transport modes have been described for OCTN2, either murine or human, namely a Na^+^-carnitine symport and a Na^+^-dependent carnitine/acylcarnitine antiport (Ohashi et al., 2001; Ohnishi et al., 2003; Pochini et al., 2013). This protein also mediates the efflux of carnitine derivatives favored by their outwardly directed concentration gradient (Figure 2 and Table 1; Xiang et al., 2017). Therefore, the actual transport mechanism, i.e., symport and/or antiport is a still an open question: it can be speculated that OCTN2 may show different transport features according to isoforms and to the tissue in which it is expressed (Ohashi et al., 1999; Pochini et al., 2013); such a regulation could be ascribed also to post-translational modifications. In this respect, the N-glycosylation pathway (Filippo et al., 2011), as well as a more complicated process involving a multiprotein complex that traffics OCTN2 to the definitive location, have been described (Jurkiewicz et al., 2017). Interestingly, the interactions with the scaffold proteins PDZK1 and PDZK2 have been described as begin responsible for the regulation of both rat and human OCTN2 transport function (Kato et al., 2005; Juraszek and Nalecz, 2019). The possible transport mechanisms together with the wide tissue distribution underlie the role of OCTN2 as the major transporter responsible for the traffic of carnitine and carnitine derivatives in the intestine, distribution to several body districts and reabsorption/excretion in the kidney (Figure 2). Even though the 3D structure of OCTN2 is still not known, the molecular determinants for substrate specificity have been revealed: one ammonium and one carboxylate groups are strictly required for transport, while the distance between them is not important (Ohashi et al., 2002; Pochini et al., 2013). The esterification of the carnitine hydroxyl group is well tolerated. The wide specificity toward carnitine derivatives is very important in the regulation of their traffic and is in line with the detection of more than 100 different types of acylcarnitines in plasma and urine (Xiang et al., 2017). The extracellular Km toward carnitine, measured in different experimental systems, ranges from 8 to 80 μM depending on different species and/or tissues. This range falls within the average carnitine concentration in plasma (Indiveri et al., 2010; Pochini et al., 2013; Figure 2). The intracellular Km for carnitine, which is also important for understanding the mechanism of carnitine traffic has been measured only in proteoliposomes, an experimental system which gives access to the internal face of a transporter (Scalise et al., 2013). The internal Km value is in the millimolar range, i.e., in the average intracellular carnitine concentrations (Pochini et al., 2004). In line with the link with FAO, the regulation of OCTN2 occurs via some transcription factors which also govern other proteins connected with lipid metabolism. As an example, the peroxisome proliferator-activated receptor α (PPARα) can activate OCTN2 expression as well as the FAO (Maeda et al., 2008). Moreover, the peroxisome proliferator-activated receptor γ (PPARγ) regulates OCTN2 expression by binding to the PPAR-response element within the first intron (Qu et al., 2014). Moreover, an estrogen hormonal regulation has been reported. Indeed, a responsive element for estrogen is located in the second intron of OCTN2 (Wang et al., 2012). Finally, insulin-dependent hormonal regulation has also been reported (Stephens et al., 2006). As stated above, the key role of OCTN2 in fatty acid metabolism is testified by human pathologies caused by alterations of the transporter function (Magoulas and El-Hattab, 2012). Some of the symptoms which characterize the inherited disease are mimicked by the administration of drugs interacting with OCTN2 as off-target or by some diet regimens (Pochini et al., 2009, 2019; El-Hattab and Scaglia, 2015). It has to be stressed that chronic inflammatory conditions may be the basis for the insurgence of other pathological states, including cancer, broadening the role of OCTN2 and its main substrate, carnitine, in human pathology. In the next section, the connections between OCTN2 expression/function and lipid metabolism in cancer will be dealt with.

**TABLE 1:** Carnitine transporters involved in cancer.

	Alias	Substrates	Expression in Cancers
SLC22A5	OCTN2	- Carnitine - Carnitine derivatives - Drugs among which anti-cancer drugs such as: Imatinib, oxaliplatin, and paclitaxel	- Glioblastoma multiforme - Ovarian carcinoma - ER+ breast cancer - Colorectal cancer - Epithelial HPV-mediated-carcinoma
SLC22A4	OCTN1	- TEA - Acetylcholine - Ergothioneine - Acetylcarnitine - Choline - Drugs among which anti-cancer drugs such as: imatinib, cytarabine, camptothecin, oxaliplatin, mitoxantrone, and doxorubicin	- Several NCI-60 cancer cell lines - Sporadic colorectal cancer and malignant progression of inflammatory bowel diseases (IBDs)
SLC22A16	CT2	- Carnitine - Drugs among which anti-cancer drugs such as: doxorubicin, bleomycin, and cisplatin	- Breast cancer - Ovarian cancer - Leukemia - Lung cancer - Gut cancer - Nasopharyngeal cancer - Diffuse large cell lymphoma - HCT116 cell line
SLC6A14	ATB^0,+^	- Amino acids - Carnitine - Propionylcarnitine - L-carnitine conjugated nanoparticles (LC-PLGANPS)	- Colorectal cancer - ER+ breast cancer - Pancreatic cancer - Cervical cancer
SLC16A9	MCT9	- Carnitine - Creatine	- Adrenocortical tumors - Diffuse large B-cell lymphoma
SLC25A20	CAC	- Carnitine - Acylcarnitine	- Prostate cancer - Non-muscle invasive bladder cancer

*OCTN2, organic cation transporter novel 2; CAC, carnitine/acylcarnitine carrier.*

### Implications of OCTN2 in Cancer

To date, several studies reported a link between the altered expression of OCTN2 and cancer development and progression (Lu et al., 2008; Scalise et al., 2012; Wang et al., 2012; Elsnerova et al., 2016; Lee et al., 2016; Fink et al., 2019; Juraszek and Nalecz, 2019; Table 1). Interestingly, its expression is found up or down-regulated depending on the carbon source that the tumors use to produce energy. Lipid utilization is increased in those cancers surrounded by a lipid-rich or lipid-enriched environment (Quail and Joyce, 2013; Koundouros and Poulogiannis, 2020). As recently reviewed, OCTN2 is overexpressed in endometrial, ovarian, renal, pancreatic cancers and also in glioblastoma (GBM) even if neurons do not normally use fatty acids to derive energy (Juraszek and Nalecz, 2019). It can be speculated that, in a condition of high aggressiveness, typical of GBM, an increase of carnitine cell content is required to sustain the high energy demand of cell proliferation. The high OCTN2 expression is associated with poor overall patient survival and a siRNA-mediated OCTN2 silencing in GBM led to a loss of tumor cell viability (Fink et al., 2019). Data were confirmed by the use of an orthotopic mouse model of GBM, in which OCTN2 was inhibited by meldonium and a reduction of tumor growth was observed (Fink et al., 2019). In metastatic breast cancer ER+ (estrogen receptor positive), OCTN2 is overexpressed due to the control exerted by the estrogen signaling. This has been experimentally demonstrated by silencing the estrogen receptor in breast cancer (ER+) with consequent decrease of OCTN2 expression (Wang et al., 2012). The link with lipid metabolism has been provided by employing a siRNA for OCTN2, which lowered carnitine uptake triggering an increased formation of lipid droplets and a decreased cell proliferation. These results suggested OCTN2 as a potential therapeutic target for breast cancer ER+. However, as recently reviewed, the role of OCTN2 in breast cancer, either ER+ or ER-, is not straightforward when considering prognosis and patient survival after cancer treatment; upon a deep analysis of breast cancer data set, the existence of a threshold level of OCTN2 expression that may affect the different prognosis has been proposed (Juraszek and Nalecz, 2019). In contrast, colorectal cancer (CRC) is characterized by decreased OCTN2 expression (Juraszek and Nalecz, 2019). In good agreement, the association between an OCTN2 SNP (rs27437), down-regulating the OCTN2 expression and the CRC risk has been identified (Zou et al., 2018). The expression of OCTN2 is decreased also in epithelial HPV-mediated carcinoma. Indeed, both mRNA and protein levels were reduced in keratinocytes retrotransduced with the high-risk HPV16E6 compared to the control (Scalise et al., 2012). A similar result was also observed in CaSki, a cervical carcinoma cell line naturally infected by HPV16. In this case, the mechanism of down-regulation is linked to promoter methylation (Scalise et al., 2012; Qu et al., 2013). As other members of the SLC22 family, OCTN2 can mediate cellular uptake of oxaliplatin and other chemotherapeutic agents (Jong et al., 2011; Pochini et al., 2019; Koepsell, 2020; Kou et al., 2020). Therefore, the epigenetic modulation of OCTN2 in cancer may by exploited to increase the efficacy of anti-cancer drugs. It is reported that treatment of CRC cells with luteolin, a naturally occurring flavonoid which is an agonist of PPARγ, increased mRNA and protein expression of OCTN2 in a time- and dose-dependent manner, enhancing the intracellular accumulation of oxaliplatin (Qu et al., 2014). In this respect, OCTN2 targeted nanoparticles have been exploited for improving paclitaxel delivery (Kou et al., 2018). Furthermore, imatinib that is the first-line treatment of patients with chronic myeloid leukemia seems to be transported in cells by OCTN2 and OCTN1 (Hu et al., 2008 and Table 1). Accordingly, an association between the SNP rs2631365-TC located in the promotor region of OCTN2 and failure of imatinib treatment has been described (Jaruskova et al., 2017). Imatinib is also used in the treatment of the gastrointestinal stromal tumor. In this cancer, the failure of imatinib mainly depends on mutations of the tyrosine-protein kinase KIT and the platelet-derived growth factor receptor α (PDGFRα). Nevertheless, some patients with responsive genotype develop resistance. After analyzing 31 polymorphisms in 11 genes, it has been discovered that in presence of the C allele in SLC22A4 (OCTN1 rs1050152), and the two minor alleles (G) in SLC22A5 (OCTN2 rs2631367 and rs2631372) the time to progression was significantly improved (Angelini et al., 2013). These studies are essential to develop personalize therapy.

### General Features of OCTN1 (SLC22A4) and Implications in Cancer

Organic cation transporter OCTN1 is another member of the SLC22 family. Its gene, named SLC22A4, counts 11 exons and encodes for a protein of 551 amino acids. This transporter is strongly expressed in the kidneys and, at a lower level, in trachea, bone marrow, liver, skeletal muscle, prostate, lung, pancreas, intestine, placenta, heart, uterus, spleen, spinal cord, and neurons (Pochini et al., 2013). The functional properties of OCTN1 have been investigated using several experimental models such as intact cells, membrane vesicles, proteoliposomes and oocyte from xenopus laevis. However, the physiological substrates of OCTN1 are still matter of discussion (Pochini et al., 2019 and Table 1). In term of transport efficiency the best substrate is the synthetic compound TEA, whereas carnitine is a poor substrate (Yabuuchi et al., 1999; Tamai et al., 2004; Pochini et al., 2019). Some studies suggest that OCTN1 mediate the uptake of a mushroom metabolite named ergothioneine, which probably exerts an anti-oxidant function in tissues exposed to ROS (Gründemann et al., 2005; Halliwell et al., 2016). Other studies demonstrated that this transporter mediates a sodium-regulated acetylcholine export suggesting a role of OCTN1 in the non-neuronal cholinergic system (Pochini et al., 2012, 2016), a ubiquitous pathway that modulates several cell functions, among which inflammation (Grando et al., 2015). Choline and acetylcarnitine have been identified as additional substrates using intact cells and proteoliposome transport assays (Pochini et al., 2015, 2016). OCTN1 is expressed in different cancer cell lines (Okabe et al., 2008). It is associated with sporadic CRC in early age, and its polymorphisms, such as L503F, may help to predict malignant progression of the disease in IBD patients (Martini et al., 2012). The involvement of OCTN1 in cancer metabolism, is probably unrelated to the poor ability of this transporter to mediate carnitine transport, however, it is interesting to know that OCTN1 is involved in the uptake of anti-cancer drugs (Table 1) such as Cytarabine (Drenberg et al., 2017), Camptothecin (Zheng et al., 2016), mitoxantrone and doxorubicin (Okabe et al., 2008).

### General Features of CT2 (SLC22A16)

The plasma membrane carnitine transporter CT2 belongs to the SLC22 family as OCTN2. The gene, mapping on chromosome 6, has been identified in 2002 in two parallel studies that classified CT2 as the sixth member of the OCT subfamily (Enomoto et al., 2002; Gong et al., 2002). One of the two reports has been conducted using healthy tissues highlighting the very narrow CT2 expression that is present almost exclusively in the plasma membrane of Sertoli cells of the testis and in the luminal membrane of epididymal cells (Koepsell, 2013). On the contrary, the other work has been performed using leukemia cells showing that CT2 is indeed expressed in cancers. In terms of functional characterization, human CT2 can transport L-carnitine with a Km of 20.3 μM. The CT2-mediated transport is partially sodium-dependent and shows a preference for basic pH (Enomoto et al., 2002 and Table 1). Apart from this preliminary characterization, no other information is available and its physiological role remains uncertain. Given its peculiar tissue distribution, a role in the sperm maturation occurring in epididymal lumen has been proposed. The sperm maturation requires a high amount of carnitine that is accumulated in epididymal cells from blood, thanks to OCTN2 (Figure 2). Then, carnitine is released in the epididymal lumen via CT2 (Jeulin and Lewin, 1996; Enomoto et al., 2002; Cotton et al., 2010) where its measured concentration reaches 50–100 mM (Figure 2). In good agreement with this role, carnitine supply to patients with asthenozoospermia results in an increase of sperm motility. Concerning the regulation, CT2 seems to respond to progesterone stimuli (Sato et al., 2007); in line with this, CT2 is expressed at a higher level during the decidualization of the endometrium that is characterized by high progesterone levels (Sato et al., 2007; Tsai et al., 2014; Liang et al., 2018).

### Implications of CT2 in Cancer

Unlike the restricted expression in healthy tissue, CT2 is largely expressed in several cancer types also originating from tissues that normally do not express this protein (Lal et al., 2007; Ota et al., 2007; Bray et al., 2010; Sagwal et al., 2018; Zhang et al., 2019 and Table 1). This denotes a profound rewiring of cancer cells concerning the carnitine linked metabolism. As an example, it is reported that the acute myeloid leukemia (AML) is strictly dependent on FAO. Knocking-down CT2 reduces the viability of tumors (Wu et al., 2015). Moreover, a recent study conducted using data of over 300 patients in 10 years, linked the up-regulation of CT2 with poor survival of gut cancer patients (Zhao et al., 2018). For the above-mentioned reasons, CT2 is considered a druggable target and has also been exploited for drug delivery. Many authors report this capacity to recognize and transport several types of anti-cancer drugs such as doxorubicin with high affinity (Okabe et al., 2005). In line with this, by using whole-exome sequencing on diffuse large B-cell lymphoma patients, a loss of the chromosomic region including CT2 gene in half of the patients has been shown. These patients experienced a lack of remission or early relapse at 24 months after diagnosis; this phenomenon has been linked with the increased resistance at doxorubicin treatment due to the lack of CT2 in the cell membrane that, in turn, impairs drug entry into cancer cells (Novak et al., 2015). The doxorubicin accumulation seems to be at the basis of the positive effects of the innovative anti-neoplastic treatment known as cold physical plasma used on different melanoma cell lines. This therapeutic approach is based on a gas treatment that induces the production of ROS/RNS able to specifically target cancer cells; interestingly, upon cold physical plasma treatment, the expression of CT2 is augmented explaining the parallel increase in doxorubicin accumulation (Sagwal et al., 2018). CT2 is also involved in the intracellular accumulation of bleomycin in human testicular cancer cells, which are sensitive to this drug (Table 1). In the same study, the human colon carcinoma cell line HCT116 was revealed to be less sensitive to the bleomycin treatment, in line with the very low expression of CT2 and with the metabolism based on glycolysis rather than on FAO (Aouida et al., 2010). Finally, cisplatin is also recognized as substrate by CT2: higher levels of CT2 expression in lung cancer was correlated to increased cellular uptake of cisplatin (Table 1). While, down-regulation of the transporter directly confers resistance against the drug, via decreasing the intracellular platinum concentration (Kunii et al., 2015).

### General Features of ATB^0,+^ (SLC6A14)

ATB^0,+^ is a plasma membrane transporter belonging to the SLC6 family that includes amino acid transporters, neurotransmitter transporters, and osmolyte transporters (Pramod et al., 2013). The gene has been annotated in the human genome on the chromosome X, it is composed of 13 exons encoding only one splice variant, and has been cloned in 1999 (Sloan and Mager, 1999). ATB^0,+^ mediates the transport of all proteogenic amino acids except for aspartate and glutamate. Intriguingly, ATB^0,+^ also recognizes carnitine as a substrate, even though with a lower affinity (Km in the millimolar range) with respect to the amino acid substrates (Sloan and Mager, 1999; Bode, 2001) and to OCTN2 (Table 1). The transport reaction catalyzed by ATB^0,+^ occurs as a co-transport of 2Na^+^ and 1Cl^–^ being highly concentrative and sensitive to membrane potential (Nakanishi et al., 2001). In contrast with OCTN2, ATB^0,+^ does not transport carnitine derivatives except for propionylcarnitine (Nakanishi et al., 2001). The subcellular localization of ATB^0,+^ is in the apical side of lung and intestine epithelia (Figure 2). In this location, the transporter is exposed to the external environment, and then, to bacteria (Rotoli et al., 2020). In agreement with this, ATB^0,+^ is up-regulated in inflammatory states, such as ulcerative colitis and Crohn’s disease (Broer and Fairweather, 2018). The interplay of ATB^0,+^ with lung bacteria has been suggested as responsible for modulating *Pseudomonas aeruginosa* attachment to human bronchial epithelial cells with consequent effects on lung disease severity in cystic fibrosis (Di Paola et al., 2017).

### Implications of ATB^0,+^ in Cancer

In the last decade, the over-expression of ATB^0,+^ in several human cancers became a hallmark of this pathology similar to what it is described for other plasma membrane transporters (Bhutia and Ganapathy, 2016 and Table 1). This has been linked particularly with the ability of ATB^0,+^ to mediate high capacity transport of amino acids (Gupta et al., 2006; Ruffin et al., 2020) making this transporter a druggable target (Karunakaran et al., 2011). However, in light of its ability to mediate uptake of carnitine, it cannot be excluded that the over-expression of ATB^0,+^ is also responsible for providing this essential co-factor to those cells in which FAO is responsible for deriving energy required for cell growth and progression. ATB^0,+^ represents also a way to deliver drugs; in this respect, studies conducted employing fluorescently labeled nanoparticles, showed the co-localization of OCTN2 and ATB^0,+^ which are responsible for mediating the cellular uptake of L-carnitine conjugated nanoparticles (LC-PLGANPs) loaded with 5-fluorouracil (Table 1). As the expression levels of OCTN2 and ATB^0,+^ are higher in colon cancer cells than in normal colon cells, LC-PLGA NPs can be used to deliver chemotherapeutic drugs selectively to cancer cells for colon cancer therapy. These findings indicate a great potential of the dual-targeting strategy (Kou et al., 2017).

### General Features of MCT9 (SLC16A9) and Implications in Cancer

MCT9 is a plasma membrane transporter belonging to the monocarboxylic acid transporter family SLC16 that counts 14 members. The gene has been identified in 1999 and annotated on chromosome 10 (Halestrap and Price, 1999). The majority of SLC16 proteins usually catalyze proton-linked transport of monocarboxylate substrates like lactic acid and pyruvic acid, while some members recognize aromatic amino acids and thyroid hormone (Halestrap and Wilson, 2012; Halestrap, 2013). In contrast with the other members of SLC16 family, MCT9 is responsible for carnitine, but also creatine, transport presumably at the basolateral membrane of enterocytes (Figure 2 and Table 1). In good agreement with this role, genome-wide association studies correlated SNPs occurring at the level of SLC16A9 gene, with carnitine, propionylcarnitine and urate levels in serum (Kolz et al., 2009; Demirkan et al., 2015). The transport mechanism of MCT9, described in *Xenopus* oocytes, revealed to be Na^+^ and pH-independent (Futagi et al., 2020). An important link with carnitine derives from chronic hepatitis B patients. HBsAg loss in this patient, which is the most important step forward to the recovery from disease, is strictly related to polymorphisms of SLC16A9 gene and, consequently to the carnitine levels (Jansen et al., 2014). A possible implication of MCT9 in some cancer types was reported: the transporter seems to be a good diagnostic target to distinguish benign from malignant adrenocortical tumors (Fernandez-Ranvier et al., 2008). Moreover, SLC16A9 is highly expressed in diffuse large B-cell lymphoma (Lim et al., 2015; Table 1).

### General Features of CAC (SLC25A20)

Carnitine/acylcarnitine carrier belongs to the SLC25 family also called mitochondrial carrier family; its gene maps on chromosome 3 (Palmieri, 2013). It is well assessed that the CAC catalyzes the entry of acylcarnitines into the mitochondrial matrix in exchange for free carnitine (Figure 3 and Table 1). In mammals, CAC can accept acylcarnitines with acyl chains of various lengths from 2 to 18 carbon atoms as substrates, but it showed a higher affinity for acylcarnitines with longer carbon chains (Indiveri et al., 2011). CAC is crucial in the control of the influx of acyl units into mitochondria and hence of FAO (Figures 2, 3). CAC transport activity is finely regulated by post-translational modifications. It has been demonstrated that acetylation of some lysine of CAC inhibits the uptake of carnitine into mitochondria and hence negatively affect FAO (Giangregorio et al., 2017). Interestingly, the citrate carrier (SLC25A1), involved in fatty acid synthesis (Figure 2), is activated by acetylation (Palmieri et al., 2015). CAC is extremely sensitive to the redox state of the cell. In particular, the oxidation state of two out of six cysteines controls the CAC via the formation of a disulfide bridge that switches off the transporter activity. In this respect, it is interesting that gasotransmitters, such as NO or H_2_S, can act on the same two cysteine residues modulating the CAC function (Giangregorio et al., 2016; Tonazzi et al., 2017). It is reported that the expression of CAC is up-regulated by statins, fibrates and retinoic acid. As for OCTN2 and several other enzymes involved in FAO, also the expression of CAC is regulated by PPAR-α. While other reports exclude that CAC could be regulated by PPAR factors (Indiveri et al., 2011).

### Implications of CAC in Cancer

To date, the correlation between CAC and cancer has received little attention, and only a few studies have reported a link between the altered expression of CAC and cancer (Table 1). As previously described, prostate cancer cells are more prone to FA utilization than normal prostate cells. A great contribution to FAO deregulation is due to the down-regulation of the same microRNAs that target CPT1A, CAC, and CAT. In particular, miR-129-5p, which shows an aberrant expression level in prostate cancer, seems to regulate the CAC expression. Forced expression of these miRNAs in prostate cancer cells, PC3 and LNCaP, results in reduced expression of CPT1A, CAC, and CAT, and hence, negatively affects FAO. This has, as a consequence, the interference with the adaptive metabolic reprogramming in prostate cancer cells (Valentino et al., 2017). Moreover, another microRNA, that is miR-212, has an aberrant expression in prostate cancer and has been shown to directly target CAC (Soni et al., 2014). These observations suggested the mitochondrial carnitine system as a potentially druggable pathway for prevention and treatment of prostate cancer (Valentino et al., 2017). Furthermore, significant alterations in the carnitine/acylcarnitine pathway were detected in bladder cancer patients. In patients with non-muscle invasive bladder cancer, the expression of CAC was significantly down-regulated compared to normal bladder tissues. A similar result was achieved also for CPT1B, CPT1C, and CAT (Kim et al., 2016). CAC seems to be a target of a first-in-class treatment for the precancerous skin condition actinic keratosis. The diterpenoid ester ingenol mebutate (IngMeb) induces cell death causing mitochondrial dysfunction and local inflammatory response. A photoreactive analog of IngMeb together with quantitative proteomic experiments were used to discover targets of IngMeb in human cancer cell lines and primary human keratinocytes. Among others, CAC results as the most prominent target of IngMeb. This drug impairs FAO through the inhibition of CAC transport activity and this can explain, at least in part, the IngMeb pharmacological mechanism of action (Parker et al., 2017). Another intriguing observation is that a relationship between the composition of the cardiolipin, the most abundant phospholipid of the inner mitochondrial membrane, and cancer cell proliferation has been found. Cardiolipin is made of a central glycerol backbone carrying four acyl groups. The nature of these acyl groups seems to be difference between cancer and normal cells (Kiebish et al., 2008). The link between the acyl composition of cardiolipin and CAC lies in the fact that this special lipid is essential for the transport activity of CAC (Tonazzi et al., 2015). If the up- or down-regulation of CAC causes an increase or decrease of mitochondrial carnitine remains to be established. It is plausible that the CAC mostly influences the rate of translocation of acyl moiety to the mitochondrial matrix. Indeed, a direct link between the activity of the CAC and the β-oxidation rate has been proposed (Indiveri et al., 2011; Tonazzi et al., 2015).

## Conclusion

The maintenance of carnitine homeostasis is crucial for cell life in physiological and pathological conditions in which FAO occurs at a high rate. Carnitine, indeed, plays the key role of shuttling acyl groups through intracellular membranes for FAO. Several human cancers rely on FAO for their development and progression to malignancy. Furthermore, carnitine is crucial in the regulation of the acyl-CoA/CoA balance, which in turn, modulates the carbohydrate and the lipid metabolisms. The carnitine and acylcarnitine traffic could not occur without the concerted activity of dedicated membrane transporters localized at the cell surface and in intracellular organelles. In line with the depicted scenario, derangements of the transporters regulating carnitine traffic and hence, carnitine homeostasis was found in several human cancers. For this reason, these proteins represent potential targets for anti-cancer therapy and should be added to the existing list, including sugar and amino acid transporters, which are already considered druggable targets. In particular, changes in the expression/function of OCTN2 and CT2 have been observed in human cancers indicating that cell supply of carnitine is strictly regulated during cancer development and that chemical KO of these proteins may serve as a strategy to impair energy production from FAO. Curiously, CT2 that, in normal conditions, has a very narrow and specific tissue distribution, becomes widely expressed in cancers even originating from tissues in which CT2 is normally not present, further highlighting the carnitine role in human cancers. The appearance of CT2 in cancers is shared with another plasma membrane transporter responsible for regulating the traffic of essential amino acids, that is LAT1 (SLC7A5; Scalise et al., 2018). Noteworthy, these two membrane transporters are also responsible for drug uptake in the cell, further highlighting their role in developing novel anti-cancer approaches and/or improving those already existing. Indeed, CT2 and OCTN2 mediate the uptake of several anti-cancer drugs such as doxorubicin and oxaliplatin, respectively. Then, changes in their expression or activity may explain the ineffectiveness of some treatments and it may be exploited to improve the delivery of drugs also in the form of carnitine-derivatives.

## Author Contributions

LC contributed in collecting bibliography, in writing the manuscript, and in conceiving and creating the figures. MS contributed in writing the manuscript and in conceiving and creating the figures. TM contributed in collecting bibliography and in writing the manuscript. AT contributed in creating the figures and in the critical revision of the manuscript. NG, MG, and LP were involved in the critical revision and writing of the manuscript. CI supervised the work and wrote and revised the manuscript. All authors contributed to the article and approved the submitted version.

## Conflict of Interest

The authors declare that the research was conducted in the absence of any commercial or financial relationships that could be construed as a potential conflict of interest. The reviewer DT declared a past collaboration with one of the authors CI to the handling Editor.

## Abbreviations

ER: endoplasmic reticulum
PDH: pyruvate dehydrogenase
TCA: tricarboxylic acid
CPT1: carnitine palmitoyltransferase 1
TMAO: trimethylamine N-oxide
OCTN2: organic cation transporter novel 2
BBB: blood-brain barrier
PCD: primary carnitine deficiency
FAO: fatty acid oxidation
CPT2: carnitine palmitoyltransferase 2
CAT: carnitine acetyltransferase
INF- γ: interferon γ
TNF- α: tumor necrosis factor- α
IDH1: isocitrate dehydrogenase 1
ROS: reactive oxygen species
PPAR α: peroxisome proliferator-activated receptor α
PPAR γ: peroxisome proliferator-activated receptor γ
GBM: glioblastoma
ER+: estrogen receptor positive
CRC: colorectal cancer
OCT: organic cation transporter
IngMeb: ingenol mebutate.

## References

[B1] Almannai M. Alfadhel M. El-Hattab A. W. (2019). Carnitine inborn errors of metabolism. *Molecules* 24:3251. 10.3390/molecules24183251 31500110 PMC6766900

[B2] Angelini S. Pantaleo M. A. Ravegnini G. Zenesini C. Cavrini G. Nannini M. (2013). Polymorphisms in OCTN1 and OCTN2 transporters genes are associated with prolonged time to progression in unresectable gastrointestinal stromal tumours treated with imatinib therapy. *Pharmacol. Res.* 68 1–6. 10.1016/j.phrs.2012.10.015 23127916

[B3] Aouida M. Poulin R. Ramotar D. (2010). The human carnitine transporter SLC22A16 mediates high affinity uptake of the anticancer polyamine analogue bleomycin-A5. *J. Biol. Chem.* 285 6275–6284. 10.1074/jbc.m109.046151 20037140 PMC2825423

[B4] Bene J. Hadzsiev K. Melegh B. (2018). Role of carnitine and its derivatives in the development and management of type 2 diabetes. *Nutr. Diabetes* 8:8. 10.1038/s41387-018-0017-1 PMC5856836 29549241

[B5] Bhutia Y. D. Ganapathy V. (2016). Glutamine transporters in mammalian cells and their functions in physiology and cancer. *Biochim. Biophys. Acta* 1863 2531–2539. 10.1016/j.bbamcr.2015.12.017 26724577 PMC4919214

[B6] Bode B. P. (2001). Recent molecular advances in mammalian glutamine transport. *J. Nutr.* 131(Suppl. 9) 2475S–2485S. 11533296 10.1093/jn/131.9.2475S

[B7] Bray J. Sludden J. Griffin M. J. Cole M. Verrill M. Jamieson D. (2010). Influence of pharmacogenetics on response and toxicity in breast cancer patients treated with doxorubicin and cyclophosphamide. *Br. J. Cancer* 102 1003–1009. 10.1038/sj.bjc.6605587 20179710 PMC2844036

[B8] Bresolin N. Freddo L. Vergani L. Angelini C. (1982). Carnitine, carnitine acyltransferases, and rat brain function. *Exp. Neurol.* 78 285–292. 10.1016/0014-4886(82)90047-4 7140898

[B9] Broer S. Fairweather S. J. (2018). Amino acid transport across the mammalian intestine. *Compr. Physiol.* 9 343–373. 10.1002/cphy.c170041 30549024

[B10] Carracedo A. Cantley L. C. Pandolfi P. P. (2013). Cancer metabolism: fatty acid oxidation in the limelight. *Nat. Rev. Cancer* 13 227–232. 10.1038/nrc3483 23446547 PMC3766957

[B11] Console L. Scalise M. Giangregorio N. Tonazzi A. Barile M. Indiveri C. (2020). The link between the mitochondrial fatty acid oxidation derangement and kidney injury. *Front. Physiol.* 11:794. 10.3389/fphys.2020.00794 32733282 PMC7363843

[B12] Console L. Scalise M. Tonazzi A. Giangregorio N. Indiveri C. (2018). Characterization of Exosomal SLC22A5 (OCTN2) carnitine transporter. *Sci. Rep.* 8:3758. 10.1038/s41598-018-22170-7 PMC5830701 29491466

[B13] Cotton L. M. Rodriguez C. M. Suzuki K. Orgebin-Crist M. C. Hinton B. T. (2010). Organic cation/carnitine transporter, OCTN2, transcriptional activity is regulated by osmotic stress in epididymal cells. *Mol. Reprod. Dev.* 77 114–125. 10.1002/mrd.21122 19899138

[B14] Currie E. Schulze A. Zechner R. Walther T. C. Farese R. V. Jr. (2013). Cellular fatty acid metabolism and cancer. *Cell Metab.* 18 153–161. 10.1016/j.cmet.2013.05.017 23791484 PMC3742569

[B15] Demarquoy J. Le Borgne F. (2015). Crosstalk between mitochondria and peroxisomes. *World J. Biol. Chem.* 6 301–309. 10.4331/wjbc.v6.i4.301 26629313 PMC4657118

[B16] Demirkan A. Henneman P. Verhoeven A. Dharuri H. Amin N. van Klinken J. B. (2015). Insight in genome-wide association of metabolite quantitative traits by exome sequence analyses. *PLoS Genet.* 11:e1004835. 10.1371/journal.pgen.1004835 25569235 PMC4287344

[B17] Di Paola M. Park A. J. Ahmadi S. Roach E. J. Wu Y. S. Struder-Kypke M. (2017). SLC6A14 is a genetic modifier of cystic fibrosis that regulates *Pseudomonas aeruginosa* attachment to human bronchial epithelial cells. *mBio* 8:e02073-17. 10.1128/mBio.02073-17 29259090 PMC5736915

[B18] Drenberg C. D. Gibson A. A. Pounds S. B. Shi L. Rhinehart D. P. Li L. (2017). OCTN1 is a high-affinity carrier of nucleoside analogues. *Cancer Res.* 77 2102–2111. 10.1158/0008-5472.can-16-2548 28209616 PMC5419029

[B19] Duran M. Loof N. E. Ketting D. Dorland L. (1990). Secondary carnitine deficiency. *J. Clin. Chem. Clin. Biochem.* 28 359–363. 2199597

[B20] El-Hattab A. W. Scaglia F. (2015). Disorders of carnitine biosynthesis and transport. *Mol. Genet. Metab.* 116 107–112. 10.1016/j.ymgme.2015.09.004 26385306

[B21] Elsnerova K. Mohelnikova-Duchonova B. Cerovska E. Ehrlichova M. Gut I. Rob L. (2016). Gene expression of membrane transporters: importance for prognosis and progression of ovarian carcinoma. *Oncol. Rep.* 35 2159–2170. 10.3892/or.2016.4599 26820484

[B22] Enomoto A. Wempe M. F. Tsuchida H. Shin H. J. Cha S. H. Anzai N. (2002). Molecular identification of a novel carnitine transporter specific to human testis. Insights into the mechanism of carnitine recognition. *J. Biol. Chem.* 277 36262–36271. 10.1074/jbc.m203883200 12089149

[B23] Fernandez-Ranvier G. G. Weng J. Yeh R. F. Khanafshar E. Suh I. Barker C. (2008). Identification of biomarkers of adrenocortical carcinoma using genomewide gene expression profiling. *Arch. Surg.* 143 841–846; discussion 846. 18794420 10.1001/archsurg.143.9.841

[B24] Filippo C. A. Ardon O. Longo N. (2011). Glycosylation of the OCTN2 carnitine transporter: study of natural mutations identified in patients with primary carnitine deficiency. *Biochim. Biophys. Acta* 1812 312–320. 10.1016/j.bbadis.2010.11.007 21126579 PMC3026072

[B25] Fink M. A. Paland H. Herzog S. Grube M. Vogelgesang S. Weitmann K. (2019). L-carnitine-mediated tumor cell protection and poor patient survival associated with OCTN2 overexpression in glioblastoma multiforme. *Clin. Cancer Res.* 25 2874–2886. 10.1158/1078-0432.ccr-18-2380 30670496

[B26] Futagi Y. Narumi K. Furugen A. Kobayashi M. Iseki K. (2020). Molecular characterization of the orphan transporter SLC16A9, an extracellular pH- and Na(+)-sensitive creatine transporter. *Biochem. Biophys. Res. Commun.* 522 539–544. 10.1016/j.bbrc.2019.11.137 31784090

[B27] Ganapathy M. E. Ganapathy V. (2005). Amino acid transporter ATB0,+ as a delivery system for drugs and prodrugs. *Curr. Drug Targets Immune Endocr. Metabol. Disord.* 5 357–364. 10.2174/156800805774912953 16375689

[B28] Ganapathy V. Thangaraju M. Prasad P. D. (2009). Nutrient transporters in cancer: relevance to Warburg hypothesis and beyond. *Pharmacol. Ther.* 121 29–40. 10.1016/j.pharmthera.2008.09.005 18992769

[B29] Gao C. Catucci G. Castrignano S. Gilardi G. Sadeghi S. J. (2017). Inactivation mechanism of N61S mutant of human FMO3 towards trimethylamine. *Sci. Rep.* 7:14668. 10.1038/s41598-017-15224-9 PMC5676948 29116146

[B30] Giangregorio N. Tonazzi A. Console L. Indiveri C. (2017). Post-translational modification by acetylation regulates the mitochondrial carnitine/acylcarnitine transport protein. *Mol. Cell Biochem.* 426 65–73. 10.1007/s11010-016-2881-0 27864727

[B31] Giangregorio N. Tonazzi A. Console L. Lorusso I. De Palma A. Indiveri C. (2016). The mitochondrial carnitine/acylcarnitine carrier is regulated by hydrogen sulfide via interaction with C136 and C155. *Biochim. Biophys. Acta* 1860 20–27. 10.1016/j.bbagen.2015.10.005 26459002

[B32] Gong S. Lu X. Xu Y. Swiderski C. F. Jordan C. T. Moscow J. A. (2002). Identification of OCT6 as a novel organic cation transporter preferentially expressed in hematopoietic cells and leukemias. *Exp. Hematol.* 30 1162–1169. 10.1016/s0301-472x(02)00901-3 12384147

[B33] Grando S. A. Kawashima K. Kirkpatrick C. J. Kummer W. Wessler I. (2015). Recent progress in revealing the biological and medical significance of the non-neuronal cholinergic system. *Int. Immunopharmacol.* 29 1–7. 10.1016/j.intimp.2015.08.023 26362206

[B34] Gründemann D. Harlfinger S. Golz S. Geerts A. Lazar A. Berkels R. (2005). Discovery of the ergothioneine transporter. *Proc. Natl. Acad. Sci. U.S.A.* 102 5256–5261. 10.1073/pnas.0408624102 15795384 PMC555966

[B35] Gupta N. Prasad P. D. Ghamande S. Moore-Martin P. Herdman A. V. Martindale R. G. (2006). Up-regulation of the amino acid transporter ATB(0,+) (SLC6A14) in carcinoma of the cervix. *Gynecol. Oncol.* 100 8–13. 10.1016/j.ygyno.2005.08.016 16168467

[B36] Halestrap A. P. (2013). The SLC16 gene family – structure, role and regulation in health and disease. *Mol. Aspects Med.* 34 337–349. 10.1016/j.mam.2012.05.003 23506875

[B37] Halestrap A. P. Price N. T. (1999). The proton-linked monocarboxylate transporter (MCT) family: structure, function and regulation. *Biochem. J.* 343(Pt 2) 281–299. 10.1042/0264-6021:3430281 10510291 PMC1220552

[B38] Halestrap A. P. Wilson M. C. (2012). The monocarboxylate transporter family–role and regulation. *IUBMB Life* 64 109–119. 10.1002/iub.572 22162139

[B39] Halliwell B. Cheah I. K. Drum C. L. (2016). Ergothioneine, an adaptive antioxidant for the protection of injured tissues? A hypothesis. *Biochem. Biophys. Res. Commun.* 470 245–250. 10.1016/j.bbrc.2015.12.124 26772879

[B40] Hanahan D. Weinberg R. A. (2011). Hallmarks of cancer: the next generation. *Cell* 144 646–674. 10.1016/j.cell.2011.02.013 21376230

[B41] Hu S. Franke R. M. Filipski K. K. Hu C. Orwick S. J. de Bruijn E. A. (2008). Interaction of imatinib with human organic ion carriers. *Clin. Cancer Res.* 14 3141–3148. 10.1158/1078-0432.ccr-07-4913 18483382

[B42] Indiveri C. Iacobazzi V. Tonazzi A. Giangregorio N. Infantino V. Convertini P. (2011). The mitochondrial carnitine/acylcarnitine carrier: function, structure and physiopathology. *Mol. Aspects Med.* 32 223–233. 10.1016/j.mam.2011.10.008 22020112

[B43] Indiveri C. Pochini L. Oppedisano F. Tonazzi A. (2010). The carnitine transporter network: interactions with drugs. *Curr. Chem. Biol.* 4 108–123. 10.2174/187231310791170748

[B44] Jansen L. de Niet A. Stelma F. van Iperen E. P. van Dort K. A. Plat-Sinnige M. J. (2014). HBsAg loss in patients treated with peginterferon alfa-2a and adefovir is associated with SLC16A9 gene variation and lower plasma carnitine levels. *J. Hepatol.* 61 730–737. 10.1016/j.jhep.2014.05.004 24824278

[B45] Jaruskova M. Curik N. Hercog R. Polivkova V. Motlova E. Benes V. (2017). Genotypes of SLC22A4 and SLC22A5 regulatory loci are predictive of the response of chronic myeloid leukemia patients to imatinib treatment. *J. Exp. Clin. Cancer Res.* 36:55. 10.1186/s13046-017-0523-3 PMC5395939 28420426

[B46] Jeon S. M. Chandel N. S. Hay N. (2012). AMPK regulates NADPH homeostasis to promote tumour cell survival during energy stress. *Nature* 485 661–665. 10.1038/nature11066 22660331 PMC3607316

[B47] Jeulin C. Lewin L. M. (1996). Role of free L-carnitine and acetyl-L-carnitine in post-gonadal maturation of mammalian spermatozoa. *Hum. Reprod. Update* 2 87–102. 10.1093/humupd/2.2.87 9079406

[B48] Jong N. N. Nakanishi T. Liu J. J. Tamai I. McKeage M. J. (2011). Oxaliplatin transport mediated by organic cation/carnitine transporters OCTN1 and OCTN2 in overexpressing human embryonic kidney 293 cells and rat dorsal root ganglion neurons. *J. Pharmacol. Exp. Ther.* 338 537–547. 10.1124/jpet.111.181297 21606177

[B49] Juraszek B. Nalecz K. A. (2019). SLC22A5 (OCTN2) carnitine transporter-indispensable for cell metabolism, a Jekyll and Hyde of human cancer. *Molecules* 25:14. 10.3390/molecules25010014 31861504 PMC6982704

[B50] Jurkiewicz D. Michalec K. Skowronek K. Nalecz K. A. (2017). Tight junction protein ZO-1 controls organic cation/carnitine transporter OCTN2 (SLC22A5) in a protein kinase C-dependent way. *Biochim. Biophys. Acta Mol. Cell Res.* 1864 797–805. 10.1016/j.bbamcr.2017.02.014 28257821

[B51] Karunakaran S. Ramachandran S. Coothankandaswamy V. Elangovan S. Babu E. Periyasamy-Thandavan S. (2011). SLC6A14 (ATB0,+) protein, a highly concentrative and broad specific amino acid transporter, is a novel and effective drug target for treatment of estrogen receptor-positive breast cancer. *J. Biol. Chem.* 286 31830–31838. 10.1074/jbc.m111.229518 21771784 PMC3173074

[B52] Kato Y. Sai Y. Yoshida K. Watanabe C. Hirata T. Tsuji A. (2005). PDZK1 directly regulates the function of organic cation/carnitine transporter OCTN2. *Mol. Pharmacol.* 67 734–743. 10.1124/mol.104.002212 15523054

[B53] Kiebish M. A. Han X. Cheng H. Chuang J. H. Seyfried T. N. (2008). Cardiolipin and electron transport chain abnormalities in mouse brain tumor mitochondria: lipidomic evidence supporting the Warburg theory of cancer. *J. Lipid Res.* 49 2545–2556. 10.1194/jlr.m800319-jlr200 18703489 PMC2582368

[B54] Kim W. T. Yun S. J. Yan C. Jeong P. Kim Y. H. Lee I. S. (2016). Metabolic pathway signatures associated with urinary metabolite biomarkers differentiate bladder cancer patients from healthy controls. *Yonsei Med. J.* 57 865–871. 10.3349/ymj.2016.57.4.865 27189278 PMC4951461

[B55] Knyazev E. N. Mal’tseva D. V. Zacharyants A. A. Zakharova G. S. Zhidkova O. V. Poloznikov A. A. (2018). TNFalpha-induced expression of transport protein genes in HUVEC cells is associated with enhanced expression of transcription factor genes RELB and NFKB2 of the non-canonical NF-kappaB Pathway. *Bull. Exp. Biol. Med.* 164 757–761. 10.1007/s10517-018-4074-1 29658079

[B56] Koepsell H. (2013). The SLC22 family with transporters of organic cations, anions and zwitterions. *Mol. Aspects Med.* 34 413–435. 10.1016/j.mam.2012.10.010 23506881

[B57] Koepsell H. (2020). Organic cation transporters in health and disease. *Pharmacol. Rev.* 72 253–319. 10.1124/pr.118.015578 31852803

[B58] Koeth R. A. Wang Z. Levison B. S. Buffa J. A. Org E. Sheehy B. T. (2013). Intestinal microbiota metabolism of L-carnitine, a nutrient in red meat, promotes atherosclerosis. *Nat. Med.* 19 576–585. 10.1038/nm.3145 23563705 PMC3650111

[B59] Kolz M. Johnson T. Sanna S. Teumer A. Vitart V. Perola M. (2009). Meta-analysis of 28,141 individuals identifies common variants within five new loci that influence uric acid concentrations. *PLoS Genet.* 5:e1000504. 10.1371/journal.pgen.1000504 19503597 PMC2683940

[B60] Kou L. Hou Y. Yao Q. Guo W. Wang G. Wang M. (2018). L-Carnitine-conjugated nanoparticles to promote permeation across blood-brain barrier and to target glioma cells for drug delivery via the novel organic cation/carnitine transporter OCTN2. *Artif. Cells Nanomed. Biotechnol.* 46 1605–1616. 28974108 10.1080/21691401.2017.1384385

[B61] Kou L. Sun R. Xiao S. Cui X. Sun J. Ganapathy V. (2020). OCTN2-targeted nanoparticles for oral delivery of paclitaxel: differential impact of the polyethylene glycol linker size on drug delivery in vitro, in situ, and in vivo. *Drug Deliv.* 27 170–179. 10.1080/10717544.2019.1710623 31913724 PMC6968687

[B62] Kou L. Yao Q. Sivaprakasam S. Luo Q. Sun Y. Fu Q. (2017). Dual targeting of l-carnitine-conjugated nanoparticles to OCTN2 and ATB(0,+) to deliver chemotherapeutic agents for colon cancer therapy. *Drug Deliv.* 24 1338–1349. 10.1080/10717544.2017.1377316 28911246 PMC8241000

[B63] Koundouros N. Poulogiannis G. (2020). Reprogramming of fatty acid metabolism in cancer. *Br. J. Cancer* 122 4–22. 10.1038/s41416-019-0650-z 31819192 PMC6964678

[B64] Kunii E. Oguri T. Kasai D. Ozasa H. Uemura T. Takakuwa O. (2015). Organic cation transporter OCT6 mediates cisplatin uptake and resistance to cisplatin in lung cancer. *Cancer Chemother. Pharmacol.* 75 985–991. 10.1007/s00280-015-2723-x 25772757

[B65] Lahjouji K. Malo C. Mitchell G. A. Qureshi I. A. (2002). L-Carnitine transport in mouse renal and intestinal brush-border and basolateral membrane vesicles. *Biochim. Biophys. Acta* 1558 82–93. 10.1016/s0005-2736(01)00433-3 11750267

[B66] Lal S. Wong Z. W. Jada S. R. Xiang X. Chen Shu X. Ang P. C. (2007). Novel SLC22A16 polymorphisms and influence on doxorubicin pharmacokinetics in Asian breast cancer patients. *Pharmacogenomics* 8 567–575. 10.2217/14622416.8.6.567 17559346

[B67] Lee J. H. Zhao X. M. Yoon I. Lee J. Y. Kwon N. H. Wang Y. Y. (2016). Integrative analysis of mutational and transcriptional profiles reveals driver mutations of metastatic breast cancers. *Cell Discov.* 2:16025. 10.1038/celldisc.2016.25 PMC5004232 27625789

[B68] Liang Y. X. Liu L. Jin Z. Y. Liang X. H. Fu Y. S. Gu X. W. (2018). The high concentration of progesterone is harmful for endometrial receptivity and decidualization. *Sci. Rep.* 8:712. 10.1038/s41598-017-18643-w PMC5768702 29335465

[B69] Lim D. H. Kim W. S. Kim S. J. Yoo H. Y. Ko Y. H. (2015). Microarray gene-expression profiling analysis comparing PCNSL and non-CNS diffuse large B-cell lymphoma. *Anticancer Res.* 35 3333–3340. 26026093

[B70] Liu Y. (2006). Fatty acid oxidation is a dominant bioenergetic pathway in prostate cancer. *Prostate Cancer Prostatic. Dis.* 9 230–234. 10.1038/sj.pcan.4500879 16683009

[B71] Lombard K. A. Olson A. L. Nelson S. E. Rebouche C. J. (1989). Carnitine status of lactoovovegetarians and strict vegetarian adults and children. *Am. J. Clin. Nutr.* 50 301–306. 10.1093/ajcn/50.2.301 2756917

[B72] Longo N. Amat di San Filippo C. Pasquali M. (2006). Disorders of carnitine transport and the carnitine cycle. *Am. J. Med. Genet. C Semin. Med. Genet.* 142C 77–85. 10.1002/ajmg.c.30087 16602102 PMC2557099

[B73] Lu X. Wang Z. C. Iglehart J. D. Zhang X. Richardson A. L. (2008). Predicting features of breast cancer with gene expression patterns. *Breast Cancer Res. Treat.* 108 191–201. 10.1007/s10549-007-9596-6 18297396

[B74] Madiraju P. Pande S. V. Prentki M. Madiraju S. R. (2009). Mitochondrial acetylcarnitine provides acetyl groups for nuclear histone acetylation. *Epigenetics* 4 399–403. 10.4161/epi.4.6.9767 19755853

[B75] Maeda T. Wakasawa T. Funabashi M. Fukushi A. Fujita M. Motojima K. (2008). Regulation of OCTN2 transporter (SLC22A5) by peroxisome proliferator activated receptor alpha. *Biol. Pharm. Bull.* 31 1230–1236. 10.1248/bpb.31.1230 18520060

[B76] Maekawa S. Mori D. Nishiya T. Takikawa O. Horinouchi T. Nishimoto A. (2007). OCTN2VT, a splice variant of OCTN2, does not transport carnitine because of the retention in the endoplasmic reticulum caused by insertion of 24 amino acids in the first extracellular loop of OCTN2. *Biochim. Biophys. Acta* 1773 1000–1006. 10.1016/j.bbamcr.2007.04.005 17509700

[B77] Magoulas P. L. El-Hattab A. W. (2012). Systemic primary carnitine deficiency: an overview of clinical manifestations, diagnosis, and management. *Orphanet. J. Rare Dis.* 7:68. 10.1186/1750-1172-7-68 22989098 PMC3495906

[B78] Martini M. Ferrara A. M. Giachelia M. Panieri E. Siminovitch K. Galeotti T. (2012). Association of the OCTN1/1672T variant with increased risk for colorectal cancer in young individuals and ulcerative colitis patients. *Inflamm. Bowel Dis.* 18 439–448. 10.1002/ibd.21814 21793125

[B79] Melone M. A. B. Valentino A. Margarucci S. Galderisi U. Giordano A. Peluso G. (2018). The carnitine system and cancer metabolic plasticity. *Cell Death Dis.* 9:228. 10.1038/s41419-018-0313-7 PMC5833840 29445084

[B80] Nakanishi T. Hatanaka T. Huang W. Prasad P. D. Leibach F. H. Ganapathy M. E. (2001). Na+- and Cl–coupled active transport of carnitine by the amino acid transporter ATB(0,+) from mouse colon expressed in HRPE cells and Xenopus oocytes. *J. Physiol.* 532(Pt 2) 297–304. 10.1111/j.1469-7793.2001.0297f.x 11306651 PMC2278546

[B81] Nezu J. Tamai I. Oku A. Ohashi R. Yabuuchi H. Hashimoto N. (1999). Primary systemic carnitine deficiency is caused by mutations in a gene encoding sodium ion-dependent carnitine transporter. *Nat. Genet.* 21 91–94. 10.1038/5030 9916797

[B82] Novak A. J. Asmann Y. W. Maurer M. J. Wang C. Slager S. L. Hodge L. S. (2015). Whole-exome analysis reveals novel somatic genomic alterations associated with outcome in immunochemotherapy-treated diffuse large B-cell lymphoma. *Blood Cancer J.* 5:e346. 10.1038/bcj.2015.69 26314988 PMC4558593

[B83] Oellgaard J. Winther S. A. Hansen T. S. Rossing P. von Scholten B. J. (2017). Trimethylamine N-oxide (TMAO) as a new potential therapeutic target for insulin resistance and cancer. *Curr. Pharm. Des.* 23 3699–3712. 28641532 10.2174/1381612823666170622095324

[B84] Ohashi R. Tamai I. Inano A. Katsura M. Sai Y. Nezu J. (2002). Studies on functional sites of organic cation/carnitine transporter OCTN2 (SLC22A5) using a Ser467Cys mutant protein. *J. Pharmacol. Exp. Ther.* 302 1286–1294. 10.1124/jpet.102.036004 12183691

[B85] Ohashi R. Tamai I. Nezu Ji J Nikaido H. Hashimoto N. Oku A. (2001). Molecular and physiological evidence for multifunctionality of carnitine/organic cation transporter OCTN2. *Mol. Pharmacol.* 59 358–366. 10.1124/mol.59.2.358 11160873

[B86] Ohashi R. Tamai I. Yabuuchi H. Nezu J. I. Oku A. Sai Y. (1999). Na(+)-dependent carnitine transport by organic cation transporter (OCTN2): its pharmacological and toxicological relevance. *J. Pharmacol. Exp. Ther.* 291 778–784. 10525100

[B87] Ohnishi S. Saito H. Fukada A. Inui K. (2003). Distinct transport activity of tetraethylammonium from L-carnitine in rat renal brush-border membranes. *Biochim. Biophys. Acta* 1609 218–224. 10.1016/s0005-2736(02)00703-4 12543384

[B88] Okabe M. Szakács G. Reimers M. A. Suzuki T. Hall M. D. Abe T. (2008). Profiling SLCO and SLC22 genes in the NCI-60 cancer cell lines to identify drug uptake transporters. *Mol. Cancer Ther.* 7 3081–3091. 10.1158/1535-7163.mct-08-0539 18790787 PMC2597359

[B89] Okabe M. Unno M. Harigae H. Kaku M. Okitsu Y. Sasaki T. (2005). Characterization of the organic cation transporter SLC22A16: a doxorubicin importer. *Biochem. Biophys. Res. Commun.* 333 754–762. 10.1016/j.bbrc.2005.05.174 15963465

[B90] Ota K. Ito K. Akahira J. Sato N. Onogawa T. Moriya T. (2007). Expression of organic cation transporter SLC22A16 in human epithelial ovarian cancer: a possible role of the adriamycin importer. *Int. J. Gynecol. Pathol.* 26 334–340. 10.1097/01.pgp.0000236951.33914.1b 17581421

[B91] Palmieri E. M. Spera I. Menga A. Infantino V. Porcelli V. Iacobazzi V. (2015). Acetylation of human mitochondrial citrate carrier modulates mitochondrial citrate/malate exchange activity to sustain NADPH production during macrophage activation. *Biochim. Biophys. Acta* 1847 729–738. 10.1016/j.bbabio.2015.04.009 25917893

[B92] Palmieri F. (2013). The mitochondrial transporter family SLC25: identification, properties and physiopathology. *Mol. Aspects Med.* 34 465–484. 10.1016/j.mam.2012.05.005 23266187

[B93] Parker C. G. Kuttruff C. A. Galmozzi A. Jorgensen L. Yeh C. H. Hermanson D. J. (2017). Chemical proteomics identifies SLC25A20 as a functional target of the ingenol class of actinic keratosis drugs. *ACS Cent Sci.* 3 1276–1285. 10.1021/acscentsci.7b00420 29296668 PMC5746860

[B94] Phillips I. R. Shephard E. A. (2020). Flavin-containing monooxygenase 3 (FMO3): genetic variants and their consequences for drug metabolism and disease. *Xenobiotica* 50 19–33. 10.1080/00498254.2019.1643515 31317802

[B95] Pietrocola F. Galluzzi L. Bravo-San Pedro J. M. Madeo F. Kroemer G. (2015). Acetyl coenzyme A: a central metabolite and second messenger. *Cell Metab.* 21 805–821. 10.1016/j.cmet.2015.05.014 26039447

[B96] Pochini L. Galluccio M. Scalise M. Console L. Indiveri C. (2019). OCTN: a small transporter subfamily with great relevance to human pathophysiology, drug discovery, and diagnostics. *SLAS Discov.* 24 89–110. 10.1177/2472555218812821 30523710

[B97] Pochini L. Oppedisano F. Indiveri C. (2004). Reconstitution into liposomes and functional characterization of the carnitine transporter from renal cell plasma membrane. *Biochim. Biophys. Acta* 1661 78–86. 10.1016/j.bbamem.2003.12.001 14967477

[B98] Pochini L. Scalise M. Di Silvestre S. Belviso S. Pandolfi A. Arduini A. (2016). Acetylcholine and acetylcarnitine transport in peritoneum: role of the SLC22A4 (OCTN1) transporter. *Biochim. Biophys. Acta* 1858 653–660. 10.1016/j.bbamem.2015.12.026 26724204

[B99] Pochini L. Scalise M. Galluccio M. Amelio L. Indiveri C. (2011). Reconstitution in liposomes of the functionally active human OCTN1 (SLC22A4) transporter overexpressed in *Escherichia coli*. *Biochem. J.* 439 227–233. 10.1042/bj20110544 21726197

[B100] Pochini L. Scalise M. Galluccio M. Indiveri C. (2013). OCTN cation transporters in health and disease: role as drug targets and assay development. *J. Biomol. Screen* 18 851–867. 10.1177/1087057113493006 23771822

[B101] Pochini L. Scalise M. Galluccio M. Pani G. Siminovitch K. A. Indiveri C. (2012). The human OCTN1 (SLC22A4) reconstituted in liposomes catalyzes acetylcholine transport which is defective in the mutant L503F associated to the Crohn’s disease. *Biochim. Biophys. Acta* 1818 559–565. 10.1016/j.bbamem.2011.12.014 22206629

[B102] Pochini L. Scalise M. Indiveri C. (2009). Inactivation by omeprazole of the carnitine transporter (OCTN2) reconstituted in liposomes. *Chem. Biol. Interact.* 179 394–401. 10.1016/j.cbi.2008.10.052 19041296

[B103] Pochini L. Scalise M. Indiveri C. (2015). Immuno-detection of OCTN1 (SLC22A4) in HeLa cells and characterization of transport function. *Int. Immunopharmacol.* 29 21–26. 10.1016/j.intimp.2015.04.040 25937167

[B104] Pramod A. B. Foster J. Carvelli L. Henry L. K. (2013). SLC6 transporters: structure, function, regulation, disease association and therapeutics. *Mol Aspects Med.* 34 197–219. 10.1016/j.mam.2012.07.002 23506866 PMC3602807

[B105] Qu Q. Qu J. Guo Y. Zhou B. T. Zhou H. H. (2014). Luteolin potentiates the sensitivity of colorectal cancer cell lines to oxaliplatin through the PPARgamma/OCTN2 pathway. *Anticancer Drugs* 25 1016–1027. 10.1097/cad.0000000000000125 25075794

[B106] Qu Q. Qu J. Zhan M. Wu L. X. Zhang Y. W. Lou X. Y. (2013). Different involvement of promoter methylation in the expression of organic cation/carnitine transporter 2 (OCTN2) in cancer cell lines. *PLoS One* 8:e76474. 10.1371/journal.pone.0076474 24146874 PMC3797819

[B107] Qu Q. Zeng F. Liu X. Wang Q. J. Deng F. (2016). Fatty acid oxidation and carnitine palmitoyltransferase I: emerging therapeutic targets in cancer. *Cell Death Dis.* 7:e2226. 10.1038/cddis.2016.132 27195673 PMC4917665

[B108] Quail D. F. Joyce J. A. (2013). Microenvironmental regulation of tumor progression and metastasis. *Nat. Med.* 19 1423–1437. 10.1038/nm.3394 24202395 PMC3954707

[B109] Ramsay R. R. Arduini A. (1993). The carnitine acyltransferases and their role in modulating acyl-CoA pools. *Arch. Biochem. Biophys.* 302 307–314. 10.1006/abbi.1993.1216 8489235

[B110] Rebouche C. J. (2004). Kinetics, pharmacokinetics, and regulation of L-carnitine and acetyl-L-carnitine metabolism. *Ann. N. Y. Acad. Sci.* 1033 30–41. 10.1196/annals.1320.003 15591001

[B111] Rebouche C. J. Seim H. (1998). Carnitine metabolism and its regulation in microorganisms and mammals. *Annu. Rev. Nutr.* 18 39–61. 10.1146/annurev.nutr.18.1.39 9706218

[B112] Rohrig F. Schulze A. (2016). The multifaceted roles of fatty acid synthesis in cancer. *Nat. Rev. Cancer* 16 732–749. 10.1038/nrc.2016.89 27658529

[B113] Roncal C. Martinez-Aguilar E. Orbe J. Ravassa S. Fernandez-Montero A. Saenz-Pipaon G. (2019). Trimethylamine-N-Oxide (TMAO) predicts cardiovascular mortality in peripheral artery disease. *Sci. Rep.* 9:15580. 10.1038/s41598-019-52082-z PMC6821861 31666590

[B114] Rose E. C. di San Filippo C. A. Ndukwe Erlingsson U. C. Ardon O. Pasquali M. Longo N. (2012). Genotype-phenotype correlation in primary carnitine deficiency. *Hum. Mutat.* 33 118–123. 10.1002/humu.21607 21922592 PMC3240685

[B115] Rotoli B. M. Visigalli R. Barilli A. Ferrari F. Bianchi M. G. Di Lascia M. (2020). Functional analysis of OCTN2 and ATB0,+ in normal human airway epithelial cells. *PLoS One* 15:e0228568. 10.1371/journal.pone.0228568 32027707 PMC7004352

[B116] Ruffin M. Mercier J. Calmel C. Mesinele J. Bigot J. Sutanto E. N. (2020). Update on SLC6A14 in lung and gastrointestinal physiology and physiopathology: focus on cystic fibrosis. *Cell Mol. Life Sci.* 77 3311–3323. 10.1007/s00018-020-03487-x 32166393 PMC7426304

[B117] Sagwal S. K. Pasqual-Melo G. Bodnar Y. Gandhirajan R. K. Bekeschus S. (2018). Combination of chemotherapy and physical plasma elicits melanoma cell death via upregulation of SLC22A16. *Cell Death Dis.* 9:1179. 10.1038/s41419-018-1221-6 PMC6281583 30518936

[B118] Sato N. Ito K. Onogawa T. Akahira J. Unno M. Abe T. (2007). Expression of organic cation transporter SLC22A16 in human endometria. *Int. J. Gynecol. Pathol.* 26 53–60. 10.1097/01.pgp.0000225845.67245.b3 17197897

[B119] Scalise M. Galluccio M. Accardi R. Cornet I. Tommasino M. Indiveri C. (2012). Human OCTN2 (SLC22A5) is down-regulated in virus- and nonvirus-mediated cancer. *Cell Biochem. Funct.* 30 419–425. 10.1002/cbf.2816 22374795

[B120] Scalise M. Galluccio M. Console L. Pochini L. Indiveri C. (2018). The human SLC7A5 (LAT1): the intriguing histidine/large neutral amino acid transporter and its relevance to human health. *Front. Chem.* 6:243. 10.3389/fchem.2018.00243 29988369 PMC6023973

[B121] Scalise M. Pochini L. Giangregorio N. Tonazzi A. Indiveri C. (2013). Proteoliposomes as tool for assaying membrane transporter functions and interactions with xenobiotics. *Pharmaceutics* 5 472–497. 10.3390/pharmaceutics5030472 24300519 PMC3836619

[B122] Seth P. Wu X. Huang W. Leibach F. H. Ganapathy V. (1999). Mutations in novel organic cation transporter (OCTN2), an organic cation/carnitine transporter, with differential effects on the organic cation transport function and the carnitine transport function. *J. Biol. Chem.* 274 33388–33392. 10.1074/jbc.274.47.33388 10559218

[B123] Shekhawat P. S. Yang H. S. Bennett M. J. Carter A. L. Matern D. Tamai I. (2004). Carnitine content and expression of mitochondrial beta-oxidation enzymes in placentas of wild-type (OCTN2(+/+)) and OCTN2 Null (OCTN2(−/−)) Mice. *Pediatr. Res.* 56 323–328. 10.1203/01.pdr.0000134252.02876.55 15240869

[B124] Sloan J. L. Grubb B. R. Mager S. (2003). Expression of the amino acid transporter ATB 0+ in lung: possible role in luminal protein removal. *Am J. Physiol. Lung. Cell Mol. Physiol.* 284 L39–L49. 12388375 10.1152/ajplung.00164.2002

[B125] Sloan J. L. Mager S. (1999). Cloning and functional expression of a human Na(+) and Cl(-)-dependent neutral and cationic amino acid transporter B(0+). *J. Biol. Chem.* 274 23740–23745. 10.1074/jbc.274.34.23740 10446133

[B126] Soni M. S. Rabaglia M. E. Bhatnagar S. Shang J. Ilkayeva O. Mynatt R. (2014). Downregulation of carnitine acyl-carnitine translocase by miRNAs 132 and 212 amplifies glucose-stimulated insulin secretion. *Diabetes.* 63 3805–3814. 10.2337/db13-1677 24969106 PMC4207388

[B127] Stanley C. A. Hale D. E. Berry G. T. Deleeuw S. Boxer J. Bonnefont J. P. (1992). Brief report: a deficiency of carnitine-acylcarnitine translocase in the inner mitochondrial membrane. *N. Engl. J. Med.* 327 19–23. 10.1056/nejm199207023270104 1598097

[B128] Stephens F. B. Constantin-Teodosiu D. Laithwaite D. Simpson E. J. Greenhaff P. L. (2006). Insulin stimulates L-carnitine accumulation in human skeletal muscle. *FASEB J.* 20 377–379. 10.1096/fj.05-4985fje 16368715

[B129] Su X. Abumrad N. A. (2009). Cellular fatty acid uptake: a pathway under construction. *Trends Endocrinol. Metab.* 20 72–77. 10.1016/j.tem.2008.11.001 19185504 PMC2845711

[B130] Svensson R. U. Parker S. J. Eichner L. J. Kolar M. J. Wallace M. Brun S. N. (2016). Inhibition of acetyl-CoA carboxylase suppresses fatty acid synthesis and tumor growth of non-small-cell lung cancer in preclinical models. *Nat. Med.* 22 1108–1119. 10.1038/nm.4181 27643638 PMC5053891

[B131] Tamai I. Nakanishi T. Kobayashi D. China K. Kosugi Y. Nezu J. (2004). Involvement of OCTN1 (SLC22A4) in pH-dependent transport of organic cations. *Mol. Pharm.* 1 57–66. 10.1021/mp0340082 15832501

[B132] Tamai I. Ohashi R. Nezu J. Yabuuchi H. Oku A. Shimane M. (1998). Molecular and functional identification of sodium ion-dependent, high affinity human carnitine transporter OCTN2. *J. Biol. Chem.* 273 20378–20382. 10.1074/jbc.273.32.20378 9685390

[B133] Tamai I. Ohashi R. Nezu J. I. Sai Y. Kobayashi D. Oku A. (2000). Molecular and functional characterization of organic cation/carnitine transporter family in mice. *J. Biol. Chem.* 275 40064–40072. 10.1074/jbc.m005340200 11010964

[B134] Tonazzi A. Galluccio M. Oppedisano F. Indiveri C. (2006). Functional reconstitution into liposomes and characterization of the carnitine transporter from rat liver microsomes. *Biochim. Biophys. Acta* 1758 124–131. 10.1016/j.bbamem.2006.01.004 16483536

[B135] Tonazzi A. Giangregorio N. Console L. De Palma A. Indiveri C. (2017). Nitric oxide inhibits the mitochondrial carnitine/acylcarnitine carrier through reversible S-nitrosylation of cysteine 136. *Biochim. Biophys. Acta Bioenerg.* 1858 475–482. 10.1016/j.bbabio.2017.04.002 28438511

[B136] Tonazzi A. Giangregorio N. Console L. Indiveri C. (2015). Mitochondrial carnitine/acylcarnitine translocase: insights in structure/function relationships. Basis for drug therapy and side effects prediction. *Mini Rev. Med. Chem.* 15 396–405. 10.2174/138955751505150408142032 25910653

[B137] Tsai J. H. Chi M. M. Schulte M. B. Moley K. H. (2014). The fatty acid beta-oxidation pathway is important for decidualization of endometrial stromal cells in both humans and mice. *Biol. Reprod.* 90:34. 10.1095/biolreprod.113.113217 PMC4435064 24403548

[B138] Valentino A. Calarco A. Di Salle A. Finicelli M. Crispi S. Calogero R. A. (2017). Deregulation of MicroRNAs mediated control of carnitine cycle in prostate cancer: molecular basis and pathophysiological consequences. *Oncogene* 36 6030–6040. 10.1038/onc.2017.216 28671672

[B139] Vaz F. M. Wanders R. J. (2002). Carnitine biosynthesis in mammals. *Biochem. J.* 361(Pt 3) 417–429. 10.1042/0264-6021:3610417 11802770 PMC1222323

[B140] Wang C. Uray I. P. Mazumdar A. Mayer J. A. Brown P. H. (2012). SLC22A5/OCTN2 expression in breast cancer is induced by estrogen via a novel intronic estrogen-response element (ERE). *Breast Cancer Res. Treat.* 134 101–115. 10.1007/s10549-011-1925-0 22212555 PMC3416040

[B141] Wang S. Xu J. Zheng J. Zhang X. Shao J. Zhao L. (2020). Anti-inflammatory and antioxidant effects of Acetyl-L-Carnitine on atherosclerotic rats. *Med. Sci. Monit.* 26:e920250. 10.12659/MSM.920250 PMC6984015 31945029

[B142] Warburg O. Wind F. Negelein E. (1927). The metabolism of tumors in the body. *J. Gen. Physiol.* 8 519–530. 10.1085/jgp.8.6.519 19872213 PMC2140820

[B143] Waterman N. Bos C. J. Barendregt T. J. (1952). On fatty acid oxidation in malignant and normal livers. *Enzymologia* 15 307–312. 13033872

[B144] Wu X. Huang W. Prasad P. D. Seth P. Rajan D. P. Leibach F. H. (1999). Functional characteristics and tissue distribution pattern of organic cation transporter 2 (OCTN2), an organic cation/carnitine transporter. *J. Pharmacol. Exp. Ther.* 290 1482–1492. 10454528

[B145] Wu X. Prasad P. D. Leibach F. H. Ganapathy V. (1998). cDNA sequence, transport function, and genomic organization of human OCTN2, a new member of the organic cation transporter family. *Biochem. Biophys. Res. Commun.* 246 589–595. 10.1006/bbrc.1998.8669 9618255

[B146] Wu Y. Hurren R. MacLean N. Gronda M. Jitkova Y. Sukhai M. A. (2015). Carnitine transporter CT2 (SLC22A16) is over-expressed in acute myeloid leukemia (AML) and target knockdown reduces growth and viability of AML cells. *Apoptosis* 20 1099–1108. 10.1007/s10495-015-1137-x 25998464

[B147] Xi L. Brown K. Woodworth J. Shim K. Johnson B. Odle J. (2008). Maternal dietary L-carnitine supplementation influences fetal carnitine status and stimulates carnitine palmitoyltransferase and pyruvate dehydrogenase complex activities in swine. *J. Nutr.* 138 2356–2362. 10.3945/jn.108.095638 19022957

[B148] Xiang L. Wei J. Tian X. Y. Wang B. Chan W. Li S. (2017). Comprehensive analysis of *Acylcarnitine* species in db/db mouse using a novel method of high-resolution parallel reaction monitoring reveals widespread metabolic dysfunction induced by diabetes. *Anal. Chem.* 89 10368–10375. 10.1021/acs.analchem.7b02283 28859482

[B149] Yabuuchi H. Tamai I. Nezu J. Sakamoto K. Oku A. Shimane M. (1999). Novel membrane transporter OCTN1 mediates multispecific, bidirectional, and pH-dependent transport of organic cations. *J. Pharmacol. Exp. Ther.* 289 768–773. 10215651

[B150] Yamamoto K. Abe S. Honda A. Hashimoto J. Aizawa Y. Ishibashi S. (2020). Fatty acid beta oxidation enzyme HADHA is a novel potential therapeutic target in malignant lymphoma. *Lab. Invest.* 100 353–362. 10.1038/s41374-019-0318-6 31527828

[B151] Zhang J. Z. Wu Z. H. Cheng Q. (2019). Screening and identification of key biomarkers in nasopharyngeal carcinoma: evidence from bioinformatic analysis. *Medicine (Baltimore).* 98:e17997. 10.1097/md.0000000000017997 31770211 PMC6890310

[B152] Zhao W. Wang Y. Yue X. (2018). SLC22A16 upregulation is an independent unfavorable prognostic indicator in gastric cancer. *Future Oncol.* 14 2139–2148. 10.2217/fon-2018-0207 29698084

[B153] Zheng J. Chan T. Zhu L. Yan X. Cao Z. Wang Y. (2016). The inhibitory effects of camptothecin (CPT) and its derivatives on the substrate uptakes mediated by human solute carrier transporters (SLCs). *Xenobiotica* 46 831–840. 10.3109/00498254.2015.1129080 26744836

[B154] Zou D. Lou J. Ke J. Mei S. Li J. Gong Y. (2018). Integrative expression quantitative trait locus-based analysis of colorectal cancer identified a functional polymorphism regulating SLC22A5 expression. *Eur. J. Cancer* 93 1–9. 10.1016/j.ejca.2018.01.065 29428571

